# A Newly Identified Peripheral Duplex Anchors and Stabilizes the MALAT1 Triplex

**DOI:** 10.1021/acs.biochem.4c00156

**Published:** 2024-08-27

**Authors:** Mary N. Mwangi, Michael J. Yonkunas, Abeer A. Ageeli, Kayleigh R. McGovern-Gooch, Sevde Yilmaz, Nathan J. Baird

**Affiliations:** Department of Chemistry & Biochemistry, Saint Joseph’s University, 600 S. 43rd Street, Philadelphia, Pennsylvania 19104, United States

## Abstract

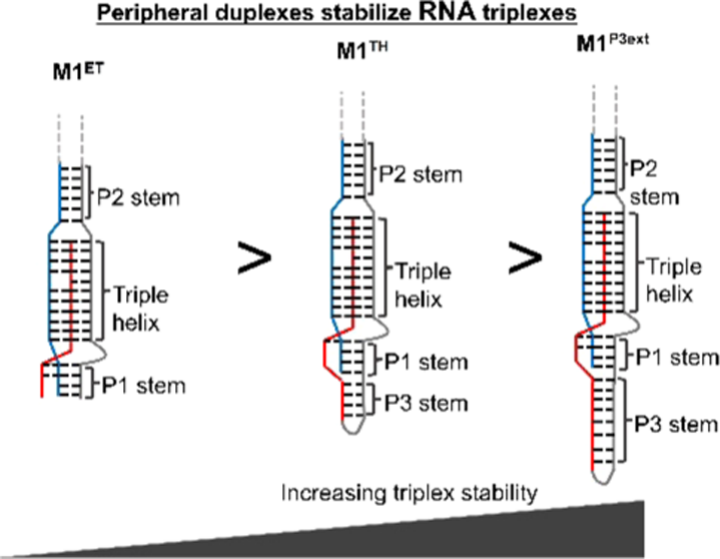

The accumulation of the 8-kb oncogenic long noncoding MALAT1 RNA in cells is dependent on the presence of a protective triple helix structure at the 3′ terminus. While recent studies have examined the functional importance of numerous base triples within the triplex and its immediately adjacent base pairs, the functional importance of peripheral duplex elements has not been thoroughly investigated. To investigate the functional importance of a peripheral linker region that was previously described as unstructured, we employed a variety of assays including thermal melting, protection from exonucleolytic degradation by RNase R, small-angle X-ray scattering, biochemical ligation and binding assays, and computational modeling. Our results demonstrate the presence of a duplex within this linker that enhances the functional stability of the triplex *in vitro*, despite its location more than 40 Å from the 3′ terminus. We present a full-length model of the MALAT1 triple helix-containing RNA having an extended rod-like structure and comprising 33 layers of coaxial stacking interactions. Taken together with recent research on a homologous triplex, our results demonstrate that peripheral elements anchor and stabilize triplexes *in vitro*. Such peripheral elements may also contribute to the formation and stability of some triple helices *in vivo*.

## Introduction

Numerous biological processes are regulated by intermolecular and intramolecular nucleic acid triple helices. Intramolecular RNA triple helices have been documented to play important roles in catalytic mechanisms, ligand binding, and protection against exonucleolytic degradation.^1^ Some RNA triple helices form through the association of an element for nuclear expression (ENE) with an A-rich tract; the ENE is a stem loop with a U-rich internal loop which forms a triple helix when paired with the A-rich tract. These structures have been identified across viral and eukaryotic long noncoding RNAs (lncRNAs).^2−4^ A well-studied example of this type of triple helix is found at the 3′ end of the metastasis-associated lung adenocarcinoma transcript 1 (MALAT1) RNA.^1,5−13^ The 3′ end of the RNA is buried within the triplex making it inaccessible to exonucleolytic degradation,^6^ resulting in the lncRNA having a half-life of 9–12 h and its accumulation in cells.^14^ In this study, as in our previous studies, we denote the MALAT1 triple helix-containing RNA as M1^TH^, which comprises the stem loop + A-rich tail.^8,15,16^ M1^TH^ is characterized by having an extended rod-like topology, which is achieved via coaxial stacking of RNA duplex structures on either side of the triplex region.^1,5−7,12,15,17,18^

Mutational analyses of the M1^TH^ using an intron-less β-globin mRNA reporter indicate transcript stabilization is significantly impacted both by the triplex region and its immediately proximal base pairs.^5^ Recently, we demonstrated that deletion of the peripheral regions, including the apical duplex or a 15-nt linker region, destabilizes the thermostability of the triplex region *in vitro.*(16) Interestingly, despite the description of the linker region as single-stranded, its deletion achieved a thermal destabilization nearly identical to the removal of seven base pairs from the duplex on the opposite side of the triplex. This study suggested an important role for the peripheral linker region in supporting the triple helix stabilization and transcript protection, despite its distal location relative to the triplex region and the RNA 3′ terminus.

To investigate the stability contribution of the linker region further, we examined the role of sequence and structure within this region. We designed several linker mutations and monitored changes in triplex stability and transcript protection *in vitro*. Our results demonstrate that reductions in thermal stability of linker mutants correlate with loss of function, leading to reduced triplex-mediated protection from exoribonucleolytic degradation by RNase R. Structural analysis by small-angle X-ray scattering (SAXS) reveals an elongated shape consistent with the formation of a helix within the linker. A rationally designed point mutant stabilizes this putative linker helix and commensurately increases functional protection from RNase R degradation. Additional biochemical analyses featuring a ligase assay and fluorescence polarization support the formation of the linker helix and stacking beneath one of the defined duplexes within M1^TH^. Finally, we present a molecular model of the full-length wild-type M1^TH^ detailing the rod-like stacking of the duplex–triplex–duplex structure. Whereas the initial crystal structure included a truncated core triple helix structure, our results reveal an elongated structure comprising 33 layers of stacking interactions that contribute to its functional stability. These results are similar to a recent study of another homologous triple helix^19^ that demonstrated significantly enhanced triplex stability upon formation of a peripheral duplex. Collectively, these results emphasize the importance of peripheral duplexes *in vitro*, which serve to anchor the triplex region and increase its stability. We suggest that peripheral anchoring duplexes may enhance naturally occurring or engineered triplexes and their functions.

## Experimental Section

### RNA Preparation

Unless otherwise noted, RNA constructs were transcribed from PCR products using T7 RNA polymerase (RNAP) as previously described.^16^ In brief, PCR primers were designed and purchased from IDT for amplification of a plasmid containing the MALAT1 triple helix sequence with an extra G (−1) at the 5′ end necessary for transcription by T7 RNAP. One RNA was designed distinctly: the construct utilized in the ligation assay (M1^truncA77^; see Figure S1) which was designed with a hammerhead sequence incorporated upstream of the 5′ end in order to generate a wild-type 5′ end following self-excision of the hammerhead sequence.^20^ All reverse primers contained two 2′-OMe nucleotides at the 5′ end in order to limit nontemplated nucleotide addition by T7 RNAP at the 3′ end of the RNA.^21^ The RNAs were purified first using preparative scale denaturing PAGE, followed by the excision of the appropriate bands and overnight electroelution (Elutrap, GE). The RNAs were ethanol precipitated, then resuspended in DEPC-treated water and stored at −20 °C.

All RNAs used in the thermal melt and small-angle X-ray scattering experiments were purified on a size-exclusion chromatography (SEC) column prior to the experiments. Unimolecular RNAs were prepared in refolding buffer (1 mM MgCl_2_, 20 mM HEPES-KOH, pH 7.4), heated at 95 °C for 2 min, snap-cooled on ice for 5 min, and allowed to equilibrate at room temperature for at least 1 h. The refolded RNAs were fractionated on an equilibrated 24-ml bed-volume ENrich SEC 70 (Bio-Rad) column in the refolding buffer. To monitor the RNA, absorbance was recorded at 260 nm. For the bimolecular RNA construct M1^ET^, the RNA was prepared in a 1:1.5 molar ratio with the excess of the short RNA (Tail) and then refolded in the refolding buffer. The RNA constructs were then fractionated on an equilibrated ENrich SEC 70 column in the refolding buffer. The two-piece RNA constructs were well separated from the excess according to the absorbance at 260 nm.

### Differential Scanning Fluorimetry (DSF)

For one DSF experiment of 40 μL final volume, RNA (540 μM) and RiboGreen dye (300 μM) were prepared in 20 mM HEPES-KOH, pH 7.4, 25 mM NaCl, and 25 mM KCl. The monovalent ion concentration was selected based on where changes in MALAT1 triplex stability are most clearly observed.^16^ A total of eight experiments were prepared and dispensed in eight columns in a 384-well plate. For magnesium titrations, a 5X stock of eight different magnesium concentrations was prepared and added to reactions for final concentrations of 0.1–1.0 mM. The RNA samples were covered with aluminum foil, centrifuged at 1,000*g* for 2 min, and incubated for 1–2 h prior to analysis.

RNA was thermally melted from 20 to 95 °C with a ramp rate of 0.015 °C/cycle using a QuantStudio 7 Flex (Thermo Fisher Scientific). An excitation filter at 470 ± 15 nm and an emission filter at 520 ± 15 nm were used for RiboGreen dye. The raw fluorescence signal and first derivative were plotted using Origin software (OriginLab). A smoothing of 70-point Fast Fourier Transform (FFT) was applied to the first derivative to aid in peak identification. The triplex melting temperatures, *T*_m,1_, and duplex melting temperatures, *T*_m,2_, peaks were identified using a peak finding algorithm in Origin.

### UV Thermal Melts

For UV melt experiments, after SEC purification, a 500–700 μL sample was prepared in the refolding buffer with a final RNA concentration of 10 μg mL^–1^, 1 mM magnesium, 25 mM NaCl, and 25 mM KCl, except for the ∼2.5 mM additional KCl introduced by the HEPES-KOH buffer. The salt conditions were chosen to mimic those of the DSF experiments.

All experiments were performed in a stoppered 1 cm quartz cuvette. Absorbance was monitored at 260 nm using an Agilent 8453 UV–vis spectrophotometer with an Agilent 89090A Peltier temperature controller. RNA was thermally melted from 20 to 95 °C with a 0.1 °C increment/cycle. Origin software (OriginLab) was used to analyze individual melting profiles and obtain the first derivatives of the absorbance as a function of temperature. The derivative data curves were smoothed using a 25-point FFT. The triplex melting temperatures, *T*_m,1_, and duplex melting temperatures, *T*_m,2_, peaks were identified using a peak finding algorithm in Origin.

### Degradation Assay

For the RNase R degradation assay, the reaction was performed as previously described.^16^ In brief, for time-course RNase R degradation reactions, 3.5 μg of one RNA was prepared in 20 mM HEPES–KOH, pH 7.4 buffer containing 0.1 mM MgCl_2_, and 25 mM KCl and 25 mM NaCl in a total volume of 70 μL. The RNA was then incubated at room temperature for 1 h. A 10 μL aliquot was removed from the RNA sample to serve as a control reaction. To the sample tube, 60 μL of RNase R mix was added that contains 6 U of RNase R (Lucigen) in 20 mM HEPES–KOH, pH 7.4, 0.1 mM MgCl_2_, and 50 mM of equimolar KCl and NaCl to have a final of 1 U of enzyme per 0.5 μg of RNA. The reactions were then incubated at 37 °C. At each time point, a 20 μL aliquot was taken from the rest of the reaction, and the RNase R activity was stopped by adding RNA loading dye (5 mM EDTA, 95% formamide); the reaction was then placed at −20 °C before being analyzed on a 6% denaturing polyacrylamide gel electrophoresis that was stained with ethidium bromide.

### Preparation of Radiolabeled RNA and Ligation of Truncated RNA by RNA Ligase 2

A 20 pmol sample of each RNA was treated with 1 unit of Shrimp Alkaline Phosphatase (SAP) in 1× SAP buffer containing 20 mM Tris-HCl, pH 8, and 10 mM MgCl_2_ (Affymetrix). The RNA was incubated at 37 °C for 2 h, followed by heat inactivation at 65 °C for 30 min, and then stored at −20 °C until further use. For the kinase reaction, 1 unit of T4 PNK was added to the tube with the addition of 5 mM DTT and 100 μCi [γ-^32^P] ATP. The reaction was incubated at 37 °C for 2 h, then the RNA was purified using 6% dPAGE, followed by the excision of the appropriate bands and the recovery of the RNA using mini-electroelution (Midi Flextube, IBI Scientific). The RNAs were ethanol precipitated overnight, then resuspended in DEPC-treated water and stored at −20 °C.

Three tubes were prepared using 100 ng of RNA with 50–100 cpm radiolabeled RNA in 1× RNA ligase 2 buffer containing 50 mM Tris-HCl, pH 7.5, 2 mM MgCl_2_, and 1 mM DTT in a 20 μL total reaction volume. Two units of the Rnl2 enzyme (NEB) was added to two of the reaction tubes. The reaction was incubated at 37 °C for 3 h, then heat inactivated at 80 °C for 5 min. For analysis of the (+) RNase R reaction, 10 units of RNase R were added to tubes containing Rnl2 and incubated at 37 °C for 1 h before running the reaction on 12% dPAGE. The gel was then dried and exposed for 1 h before imaging (Amercham Typhon, GE Healthcare). For analyzing each gel, we used Image Studio software to quantify each band. The signal of each band within one well was normalized to the total signal in that well. The ligated product has an average of 2.5 ± 0.3 times more signal intensity than the unreacted product in three independent experiments. For the RNase R reaction, a shorter band is present, representing undigested RNA due to its length. This band was taken into account while quantifying the total signal in each well (Figure S6).

### Preparation of Fluorescent Labeled RNA for Fluorescence Polarization Binding Assay

The preparation of labeled RNAs for the fluorescence polarization (FP) binding assay is described in detail elsewhere (*Mwangi and Baird, manuscript accepted for publication in MethodsX, 2024*). Briefly, unlabeled M1^A^ RNAs were purchased from Dharmacon. Modified amino nucleotide, 5-[3-aminoallyl]-2′-deoxyuridine-5′-triphosphate, AAdUTP from ThermoFisher, was attached to the 3′ terminal end of the RNAs using Klenow DNA polymerase, generating an RNA of length *n*+1. This RNA_*n*+1_, now having a 3′ terminal amine linker, was purified using denaturing gel electrophoresis. Following purification, the RNA_*n*+1_ was conjugated to the Cy3 monoreactive NHS ester dye (Cytiva) by overnight incubation. The labeled RNA was ethanol precipitated and then labeled a second time to increase the labeling efficiency. Following the second labeling reaction, the RNA was precipitated again and subsequently purified using gel electrophoresis. For this step, the labeled RNA was run on a 20% denaturing PAGE at 30 W for 2 h. The gels were then shadowed under UV light, and the band corresponding to the labeled product was excised. The gel pieces were subjected to passive elution via the crush and soak technique. The RNA was concentrated by ethanol precipitation prior to resuspension in DEPC-treated water. The concentration of the purified labeled RNA was then determined by measuring the absorbance at 260 nM using *Nanodrop* (ThermoFisher). The purified RNA was then stored at −20 °C.

### Fluorescence Polarization Binding Assay

FP measurements were collected using black 384-well polystyrene plates (Greiner BioOne 784086) in a Synergy H1Microplate Reader (Biotek) operating at room temperature using a gain setting of 100. FP data were collected using a 530 ± 25 nm excitation filter and a 590 ± 35 nm emission filter. Binding experiments were performed in replicates of *n* = 5 (unless otherwise stated) using a constant concentration of M1^A^ (tracer) and an increasing concentration of M1^B^ (see Figure S1 for structure and sequence information). All FP experiments were performed in 20 mM HEPES-KOH, pH 7.4, 25 mM NaCl, 25 mM KCl, and 0.1 mM MgCl_2_. This salt condition was chosen based on the DSF data; at these salt concentrations, the impact of the P3 helix on RNA stability is most pronounced. Each independent sample was thoroughly mixed and equilibrated at room temperature for 3 h before the FP measurement. The recorded measurement of the FP included raw parallel and perpendicular intensity data. The FP data was calculated from the buffer subtracted parallel and perpendicular intensities, as in eq 1.

Fluorescence polarization equation, where parallel intensity is represented as *I*_II_ and perpendicular intensity is represented as I_II_.
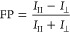
1

The change in polarization was obtained by subtracting the polarization of the tracer wells from the polarization of the RNA complexes in each well (eq 2).
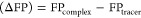
2

Plots of the change in polarization against M1^B^ concentrations were fitted using eq 3 to determine the binding affinity, *K*_D_.

Hyperbolic equation of fit for polarization data, where *S* represents the FP signal, *S*_f_ represents the FP signal of the free tracer, *S*_b_ represents the FP signal of the bound tracer in the triplex, and X is the concentration of M1^B^.
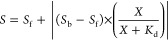
3

### Small-Angle X-ray Scattering Experiments

RNA samples for SAXS experiments were prepared as follows. Briefly, SEC purified RNA was diluted to a final concentration of 0.35 μg/μL in 120 μL in a buffer containing final concentrations of 1 mM MgCl_2_, 75 mM KCl, 75 mM NaCl, and 20 mM HEPES pH 7.5. The high ionic strength of the buffer was chosen to mimic physiological conditions.

Data was collected under continuous flow on beamline ID-12 at the Advanced Photon Source (APS) at Argonne National Laboratories, controlled and collected using beamline-specific programs and scripts. Data were collected for buffer samples and RNA samples separately. Igor Pro (WaveMetrics) was used to examine data quality. A Guinier analysis was used to calculate *R*_g_ from scattering intensities. Initial calculations of the pair-distance probability distribution, *P*(*r*), were conducted using a scan of the maximum molecule distance *D*_max_ using Python scripts^22^ and GNOM^23^ within the ATSAS analysis package (embl-hamburg.de/). *D*_max_ was determined based on a smooth plot of *P*(*r*), where *D*_max_ corresponds to an abscissa intercept at *r* = 0.

### Theoretical Scattering Calculations

Coordinates were extracted from single frames of MD simulations and imported into CRYSOL^24^ where default parameters were selected. The calculated *R*_g_ and maximum molecular diameter, excluding hydrogen volume, were recorded. Theoretical intensities generated by CRYSOL^24^ were imported into GNOM^23^ where the predicted maximum molecular diameter is input as *D*_ma*x*_. A scan of *P*(*r*), the pair distribution function, was performed at *D*_max_ ± 5 Å based on a smooth plot of *P*(*r*), where *D*_max_ corresponds to an abscissa intercept at *r* = 0. The residual sum of squares (RSS) was used to calculate the overall difference between experimental *P*(*r*) and the values predicted by theoretical scattering, as in eq 4.

Residual sum of squares equation, where the sum is over all data points in the *P*(*r*) plot and *y*_*i*_^*exp*^ and *y*_*i*_^calc^ are the *i*th experimental and calculated data points, respectively. An RSS = 0 corresponds to identical datasets.
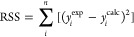
4

### M1^TH^ Linker Modeling

**Figure 1:**
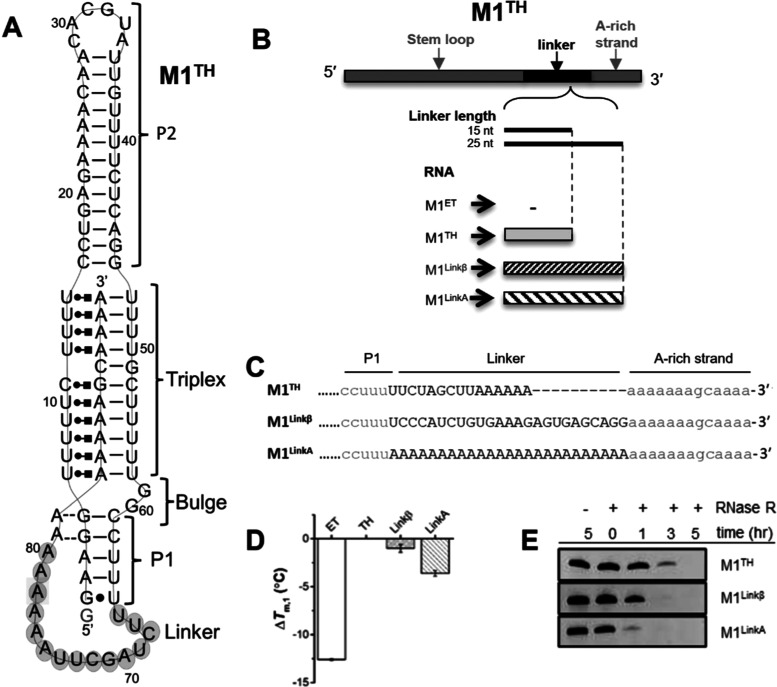
Linker mutations impact the stability and protection of the MALAT1 triple helix. (A) Secondary structure of the MALAT1 triple helix-containing RNA (M1^TH^). The linker region is indicated with gray circles. A 5′ GTP (light gray) is added to the sequence to facilitate transcription (**Methods**). (B) Representation of M1^TH^ with the linker region colored black. The role of the linker was evaluated by deletion (M1^ET^), replacement with the 25-nt linker sequence from the homologous MENβ triplex (M1^Linkβ^), and replacement with a 25-nt poly adenosine linker (M1^LinkA^). (C) The linker sequences of M1^TH^, M1^Linkβ^, and M1^LinkA^ show the difference in length and sequence. Each sequence begins at the wild-type C_61_ to include part of the P1 stem (gray), linker region (capital letters, black), and A-rich strand (gray). (D) Reduction in tertiary melting temperatures of mutants relative to M1^TH^ (Δ*T*_m,1_) for M1^ET^, M1^Linkβ^, and M1^LinkA^. All measurements were made in 1 mM MgCl_2_, 20 mM HEPES, pH 7.4, 25 mM NaCl, and 25 mM KCl. The data represents the average of three independent replicates. (E) Degradation of the mutant triplexes using 3′-5′ exoribonuclease R. Representative 6% dPAGE gels are shown for M1^TH^, M1^Linkβ^, and M1^LinkA^.

To construct a wild-type structural model of M1^TH^, three phases of modeling were implemented: the P2 apical stem, the P3 basal linker helix, and finally the attachment of P3 to the 3′ tail. First, the coordinates of nucleotides 1–21, 30–53, and 65–75 (PDB numbering) were obtained from the crystallographic triple helix stability element of *human* MALAT1, PDBID: 4PLX.^6^ Nucleotides 22–41 (wild-type sequence numbering starting at the 5′ G1, see Figure 1A) of the apical P2 were modeled using the sequence of *human* MALAT1 threaded onto an A-form RNA helical coordinate set using the RNA tools python scripts within Rosetta.^25^ The modeled P2 helix was attached to the crystallographic structure at G21 and C30 (PDB numbering) independently using 500 cycles of stepwise Monte Carlo using Rosetta.^26^ When modeling the linker helix P3, we identified an RNA via a search of the PDB for structures that contained an unpaired nucleotide flanked by two sets of base pairs within a helix in an effort to conserve the inherent structural properties of the helix. Such a structure was found in the cyclic dimeric guanosine monophosphate riboswitch (cdGR) structure (PDBID: 3IWN)^27^ and was subsequently used as a threading template. Nucleotides 41–45 and 80–83 from Chain A of PDB3IWN (cdGR structure, see Figure S8) were used as a template for M1^TH^ threading of nucleotides 66–70 and 74–77 (wild-type sequence numbering, see Figure 1A). The resulting three-nucleotide hairpin loop (G71, C72, and U73) was *de novo* modeled using the RNA_denovo module of Rosetta which performs Monte Carlo fragment assembly optimized in a knowledge-based low-resolution potential using the minimize_rna flag for steric refinement.^25,26,28^ Finally, three adenosine nucleotides (A78-A80) were constructed using PyMol′s build function (Schrodinger),^29^ connecting A77 to the crystallographic A-minor A81. The sculpting feature of PyMol (Schrodinger)^29^ was then used to optimize the geometry of nucleotides A78-A82 using residue shells with a radius and cushion of 4 Å. The final system of 93 nucleotides containing 2949 atoms was subjected to 10,000 energy minimization steps in vacuum with a nonbonded cutoff of 12 Å to relieve bond, angle, and dihedral restraints using Sander MPI in AMBER 16.^30^

## Results

### Effects of Linker Length and Sequence on the Stability of M1^TH^

The MALAT1 triple helix involves interactions between an A-rich tract and an internal U-rich loop, resulting in nine U·A-U base triples and one C^+^·G-C base triple (Figure 1A). Peripheral structural elements include two duplexes, one basal (P1) and one apical (P2), and a 15-nt basal linker region connecting P1 to the A-rich tail (Figure 1A).^5−7,17^ Previously, we demonstrated that removal of either the basal linker or half of the apical P2 duplex leads to nearly identical reductions in triplex region thermal stability (Δ*T*_m,1_ ∼ – 12.5 °C),^16^ which we refer to as *T*_m_,1, as it is the first of two melting transitions for this RNA. We therefore reasoned that the functional importance of the presumed single-stranded linker merited further investigation. We designed linker region mutants within unimolecular M1^TH^ to assess the role of linker length and sequence identity in stabilizing triple helix formation and function. To benchmark the stability and activity of our linker mutant constructs, we compare all results against the unimolecular wild-type M1^TH^ (see all secondary structures in Figure S1).

To assess the role of linker sequence identity, we first replace the 15-nt linker of M1^TH^ with the 25-nt linker from the homologous triple helix found in the multiple endocrine neoplasia-β (MENβ) lncRNA.^5^ We designate this RNA chimera as M1^linkβ^ (Figure 1B). Thermal melt analysis shows that the thermostability of the triplex region is not significantly altered (Δ*T*_m,1_ = −1 °C) (Figures 1C, S2, and Table S1). In comparison to the thermostability of the bimolecular ENE-tail (M1^ET^) construct, which lacks all linker nucleotides and significantly destabilizes the triplex region (Δ*T*_m,1_ = −12.6 °C), the M1^linkβ^ mutant results in only very minor destabilization of the triplex region melting temperature. In contrast, mutation of the linker sequence to include a string of 25 adenosine nucleotides (M1^linkA^, Figure 1B) results in moderate destabilization of the triplex region (Δ*T*_m,1_ = −3.6 °C) (Figures 1C, S2, and Table S1). Therefore, through some yet unknown manner, the linker sequence influences the stability of the MALAT1 triple helix structure.

Given the incremental thermal destabilization of the triplex region conferred by mutations within the linker, we next turned to evaluate the protection activity of the triple helix and if it is impacted by mutations in the linker region. To test this, we assessed the ability of each mutant to resist exonucleolytic degradation by RNase R *in vitro* (Figures 1D and S3), as has been used in evaluating other triple helices previously.^12,16^ Because the wild-type M1^TH^ is exceptionally resistant to degradation in near-physiological ionic conditions,^16^ we performed our analyses under conditions wherein the wild-type triple helix is degraded within 5 h (**Methods**). The M1^LinkA^ mutant was degraded almost completely within 1 h, while M1^Linkβ^ was degraded completely within 3 h (Figures 1D and S3). The distinctive and rapid degradation of M1^LinkA^ suggests an important functional role involving the linker sequence, potentially involving structural interactions within the linker.

### Inclusion of the M1^TH^ Linker Region Produces a Unique Global Conformation

**Figure 2:**
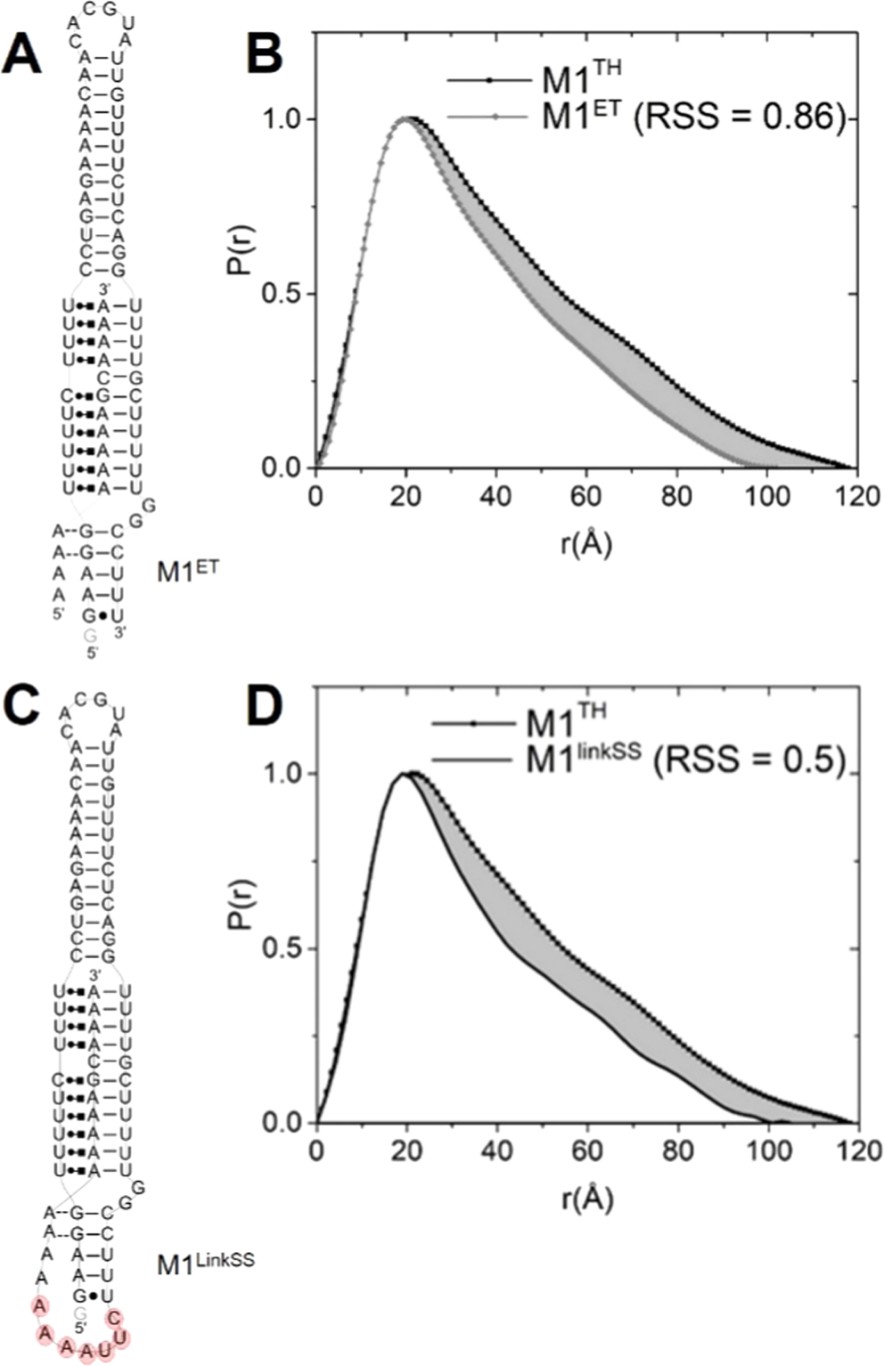
Comparison of the global shapes of M1^**TH**^, M1^ET^, and model M1^LinkSS^ using SAXS data. (A) Secondary structure of the M1^ET^ bimolecular construct, which lacks the linker region^16^ (see Figure S4A for the tertiary structure). (B) Comparing *P*(*r*) plots for M1^TH^ (black line + symbol) and M1^ET^ (gray line + symbol). The shaded region between the two data sets highlights the difference in global structure distribution between the two RNAs (RSS = 0.86). (C) Secondary structure of M1^LinkSS^, which contains a truncated linker (see Figure S4B for the tertiary structure). (**D)** Comparing the *P*(*r*) plot for the model M1^LinkSS^ (line) to M1^TH^ (black line + symbol). The shaded region between the two data sets highlights the difference in global structure distribution between the two RNAs (RSS = 0.5). All SAXS data were performed in 1 mM MgCl_2_, 75 mM NaCl, and 75 mM KCl in 20 mM HEPES, pH 7.4 buffer.

Given that the linker sequence impacts M1^TH^ thermal stability and protection against RNase R degradation, we reasoned that the linker region might form some structure that contributes to these functions. We next sought to examine the putative structure in this region using solution small-angle X-ray scattering (SAXS) (Figures 2A,B, and S4). A comparison of SAXS structural parameters for M1^TH^ and M1^ET^ RNAs affords a differential description of any putative structure within the linker. We evaluated the radius of gyration (*R*_g_), maximum intramolecular distance (*D*_max_), and probability distribution plots for these two triple helices containing RNAs, M1^TH^ and M1^ET^, in near-physiological conditions (**Methods**). Deletion of the linker results in reductions in *R*_g_ (Δ*R*_g_ = −4.1 Å) and *D*_max_ (Δ*D*_max_ = −16 Å) as expected (Table S2). The overall shape of M1^TH^ and M1^ET^ constructs was independently assessed using a plot of the probability distribution, or *P*(*r*), calculated from SAXS scattering intensities for each respective data set.^31^ The normalized *P*(*r*) describes the paired set of all distances between points within each RNA construct (Figure 2B,D). The shape of each *P*(*r*) plot is characteristic of a rod-like molecule^31^ and the difference in the *P*(*r*) plots is quantified using a residual sum of squares, or RSS (**Methods**), as an optimality criterion. The discrepancy between M1^ET^ and M1^TH^*P*(*r*) plots (Figure 2B) indicates a significant loss of pairwise structural interactions longer than 25 Å upon removal of the linker nucleotides, indicative of an overall shortened rod-like structure.

To assess the type and extent of structure within the linker nucleotides, we first evaluated the arrangement of the 9-nt linker present in the available crystal structure (PDBID: 4PLX). This 9-nt single-stranded linker projects outward to the side of the P1 helix, owing to crystal contacts with another molecule^6^ (Figure S4C). We then generated an atomic model starting from the crystal structure and including a wild-type apical P2 helix (Figures 2C and S4B) (**Methods**). This new model, M1^LinkSS^, includes the truncated single-stranded linker present in the crystal structure. Next, we calculated the theoretical SAXS scattering parameters from this model and compared them with the experimental SAXS parameters for the wild-type M1^TH^. The *R*_g_ and *D*_max_ are smaller than the experimentally determined parameters for the wild-type M1^TH^ by 2.2 and 23 Å, respectively (Table S2). Furthermore, the difference in *P*(*r*) plots between M1^TH^ and M1^LinkSS^ has RSS = 0.5, again indicating global differences in pairwise distances larger than 25 Å and an overall shortened rod-like structure. The incongruence of the calculated *R*_g_, *D*_max_, and *P*(*r*) plots (Figure 2D) between the M1^LinkSS^ model and M1^TH^ demonstrate that the structural arrangement of the truncated linker, M1^LinkSS^, does not closely approximate the arrangement of the 15-nt wild-type linker in solution (Figure 2D). These differences between M1^LinkSS^ and M1^TH^ suggest that the linker nucleotides should extend below P1, extending the *D*_*max*_ along the rod-like axis of the triplex and also leading to an increased *R*_g_ consistent with those parameters determined experimentally for M1^TH^. These comparative analyses provide strong evidence that the M1^TH^ linker region adopts an extended, ordered structure.

### Mutations within the M1^TH^ Linker Region Support the Formation of an RNA Duplex

**Figure 3:**
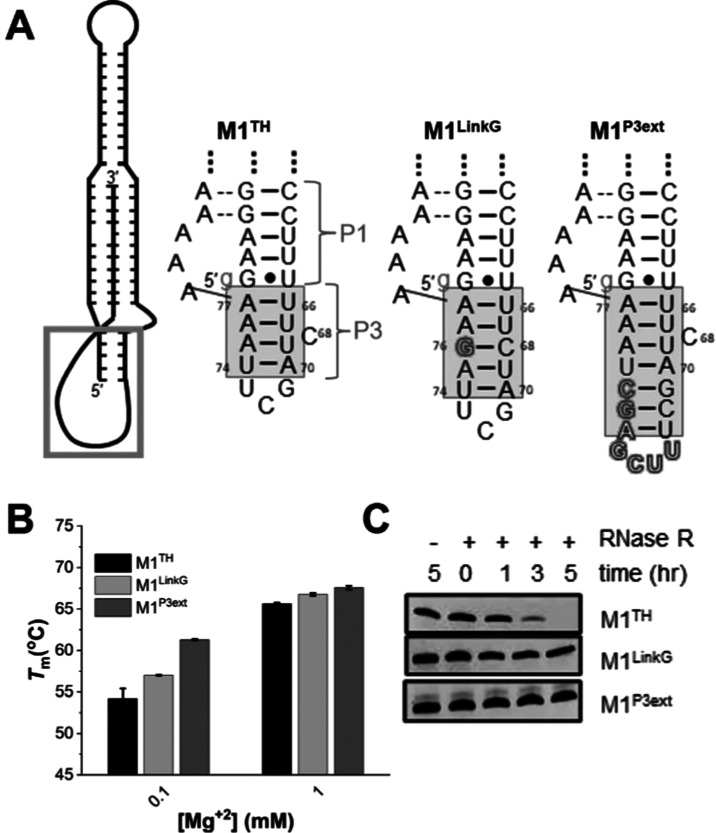
A newly identified P3 helix within the linker region. (A) Secondary structure depiction of M1^TH^ with boxed linker region. Also depicted are the linker region sequences and secondary structures in M1^TH^, M1^LinkG^ mutant, and M1^P3ext^ mutant. The M1^LinkG^ mutant inserts G after A_75_. The M1^P3ext^ has an extended P3 helix with an insertion of 7 nt (black outline). Gray rectangles highlight the proposed base-pairing regions within the P3 helix. A 5′ GTP (light gray) is added to the sequence to facilitate transcription (**Methods**). (B) Comparison of the triplex melting temperature between M1^TH^ (black), M1^LinkG^ (light gray), M1^P3ext^ (dark gray) at two MgCl_2_ concentrations in 25 mM NaCl and 25 mM KCl in 20 mM HEPES, pH 7.4. (C) RNase R degradation of M1^TH^, M1^LinkG^, and M1^P3ext^ over a 5 h time course. For M1^P3ext^ the doublet in the gel is due to an aberration during gel running; see Figure S3, where the control lanes also show a similar doublet.

Based on the SAXS results, we reasoned that the most plausible organization would include base pairing interactions within the linker region that coaxially stack with the P1 helix. Examination of the wild-type linker sequence (Figure 1A) indicates several potential base pairs: U_66_-A_77_, U_67_-A_76_, U_69_-A_75_, and A_70_-U_74_ (Figure 3A). These would comprise a 4-base pair helix with an internal 1-nucleotide bulge at C_68_. To assess the putative formation of this ordered structure within the M1^TH^ linker region, we conducted mutational analyses and a biochemical ligation experiment.

We first employed mutational analysis on the linker region to evaluate the potential structure in this region. We designed a linker mutant, M1^LinkG^ (**Methods**), with an inserted G between A_75_ and A_76_, which would base pair with C_68_ if indeed a helical arrangement is present within the linker in solution (Figure 3A). This insertion would serve both to improve the stacking interactions and increase the amount of ordered structure within the linker, putatively stacking beneath the P1 helix and adding stability to the duplex–triplex–duplex rod-like structure. To examine the impact of this mutation on triplex thermostability, we performed differential scanning fluorimetry experiments (DSF) monitoring the triplex melting transitions of M1^LinkG^ and M1^TH^ over a range of magnesium concentrations (0.1–1 mM) (Figure S5). M1^LinkG^ is more thermostable than M1^TH^ under all conditions tested, with the increased stability amplified at the low magnesium concentration (Figure 3B and Table S3). To assess whether the increased thermostability correlates with improved triplex-mediated protection from degradation, we evaluated the M1^LinkG^ mutant for its resistance (or susceptibility) to degradation by RNase R. In contrast to the M1^Linkβ^ and M1^LinkA^ linker mutants, which led to faster exonucleolytic degradation (Figures 1B,D, and S3), M1^LinkG^ is more resistant to degradation by RNase R than M1^TH^ (Figures 3C and S3). Even after 5 h, during which time M1^TH^ is completely degraded, M1^LinkG^ is not appreciably degraded. These data support the formation of a duplex structure within the linker region, which we designate as P3. The additional putative base pair, LinkG – C_68_, together with the other putative four base pairs within the linker (Figure 3A) forms an ordered helix whose location below P1 confers favorable stacking interactions with P1, giving rise to increased functional stability.

To assess the role of increased stacking interactions in triplex stability from the helical stacking of P1 to P3 duplex, we designed another construct designated M1^P3ext^. This construct contains an insertion of 7 nucleotides between U_73_ and U_74_ (Figure 3A, M1^P3ext^). The base pairing in the P3 helix of U_66_-A_77_, U_67_-A_76_, U_69_-A_75_, and A_70_-U_74_ is maintained, and three additional base pairs are added in the linker region. The P3 helix in this construct is capped with a 4-nucleotide UUCG tetraloop. We anticipated that this construct would have increased thermal stability when compared to M1^TH^ and M1^LinkG^ due to the increased stacking interaction contributed by the elongated P3 stem. A comparison of the DSF data monitored over a range of magnesium concentrations ranging from 0.1–1 mM shows that this construct is more thermostable. When compared to M1^TH^, the M1^P3ext^ construct has an increased stability of about 7 °C at 0.1 mM magnesium, 5 °C at 0.3 mM magnesium, and 4 °C at 0.6 mM magnesium (Figures 3B, S5, and Table S3). At the highest magnesium concentration tested (1 mM), we see a similar trend to what was observed for the M1^LinkG^ mutant, wherein the difference in the triplex region melting temperature, *T*_m,1_ is lowered as a result of the increased overall stabilization of the triplex region conferred by the magnesium (Figures 3B, S5, and Table S3). The stability of the M1^P3ext^ construct is further demonstrated in the RNase R degradation assay; this construct is stable even after 5 h incubation with the enzyme, whereas M1^TH^ is completely degraded within the same time period (Figures 3C and S3).

**Figure 4:**
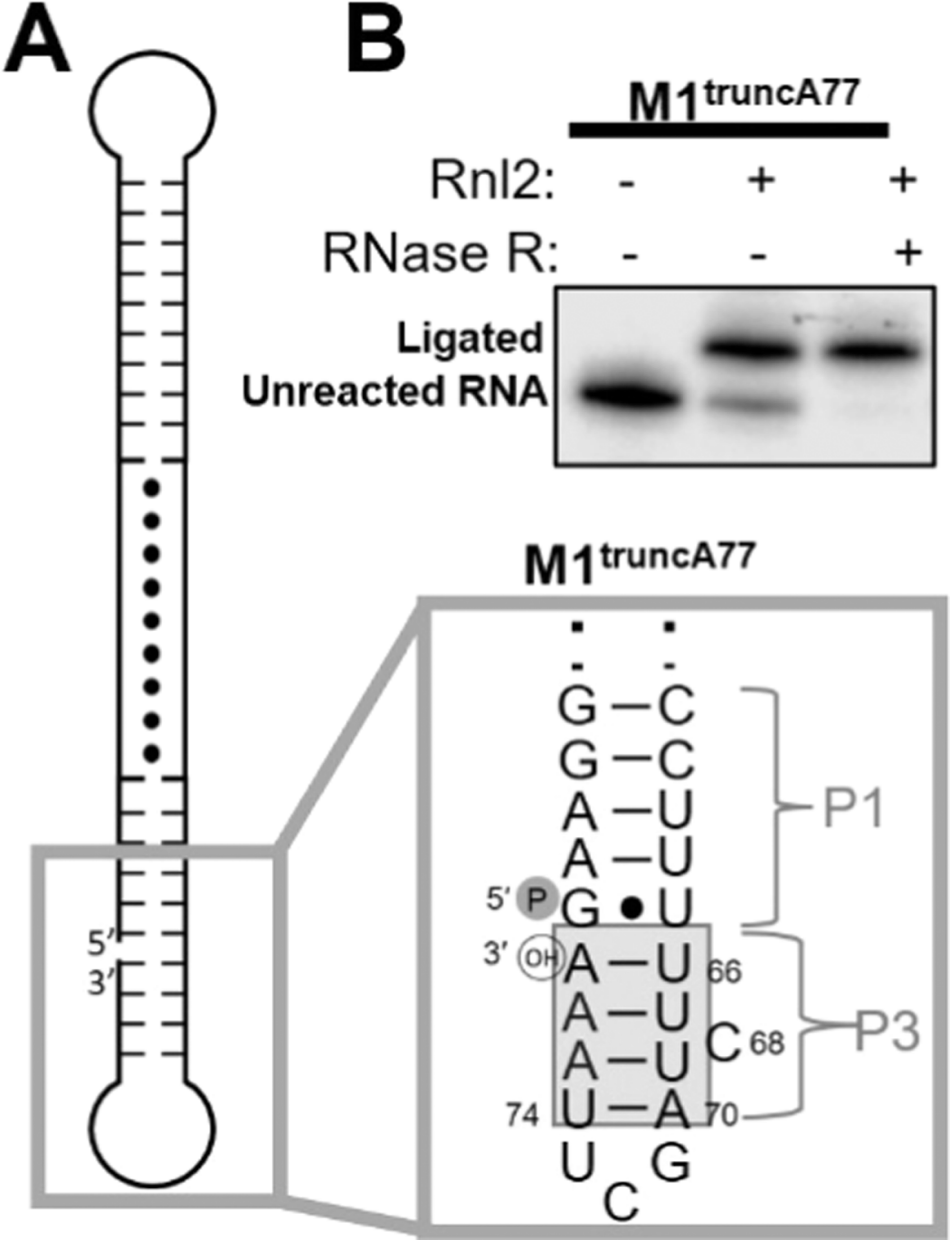
Ligation assay confirming the stacking of the P3 helix to P1. (A) Depiction of the proposed secondary structure of M1^truncA77^ construct, a truncated M1^TH^ sequence lacking the final 16 nucleotides, with a box highlighting the linker region and ligation site. Black dots indicate U·U pairs.^15^ Putative secondary structure (right) of M1^truncA77^ depicting the proximal positions of the 5′ phosphate (P) and 3′ hydroxyl (OH) groups due to the formation of the P3 helix. (B) Ligation by RNA ligase 2 (Rnl2) results in the formation of a circular RNA that migrates more slowly on a 12% denaturing gel. The total yield of the ligation is 80%, with 51% forming the main ligated circular product and 29% forming other ligation products (see Figure S6 for gel showing all the bands). The circular product is resistant to degradation by RNase R.

Based on our mutational analysis, we see that this putative P3 helix would stack beneath P1, positioning G_1_ and A_77_ proximal to each other. To directly assess this geometric arrangement, we generated a truncated construct, M1^truncA77^, whose 3′ end is positioned at A_77_ (Figure 4A). This truncated construct lacks the A-rich 3′-tail of M1^TH^ (Figure 1A), but contains the nucleotides proposed to form the P3 helix (Figure 4A). The formation of base pairs in the linker region creates a near-continuous double-stranded helix, broken only at the separation between 3′ A_77_ and the 5′ guanosine, mimicking a nicked double helix (Figure 4A). We used RNA ligase 2 (Rnl2), which specifically ligates double-stranded regions of RNA,^32^ to probe the formation of the putative double-stranded nicked helix in the linker region of the truncA77 mutant. *P^32^* radiolabeled truncA77 RNA was incubated with Rnl2, and the results were evaluated on a denaturing PAGE gel (**Methods**). A unimolecular product is observed to migrate more slowly than the unreacted control. We suggest that this band represents a circularized product (Figures 4B and S6). To assess this, following ligation, the reaction tube was subjected to exonucleolytic degradation by RNase R. Indeed, the slowly migrating band observed in the ligation reaction exhibits resistance to exonucleolytic degradation by RNase R, thereby indicating the successful formation of circular ligated products (Figures 4B and S6). In contrast, the unreacted RNA is degraded because it can dynamically adopt an unstructured conformation wherein the 3′ end is accessible to RNase R (Figures 4B and S6). Taken together, these ligation results further support the formation of the P3 duplex and its coaxial stacking with the P1 helix.

### Mutations within the M1^TH^ Linker Region Support the Formation of P1–P3 Stacking

**Figure 5:**
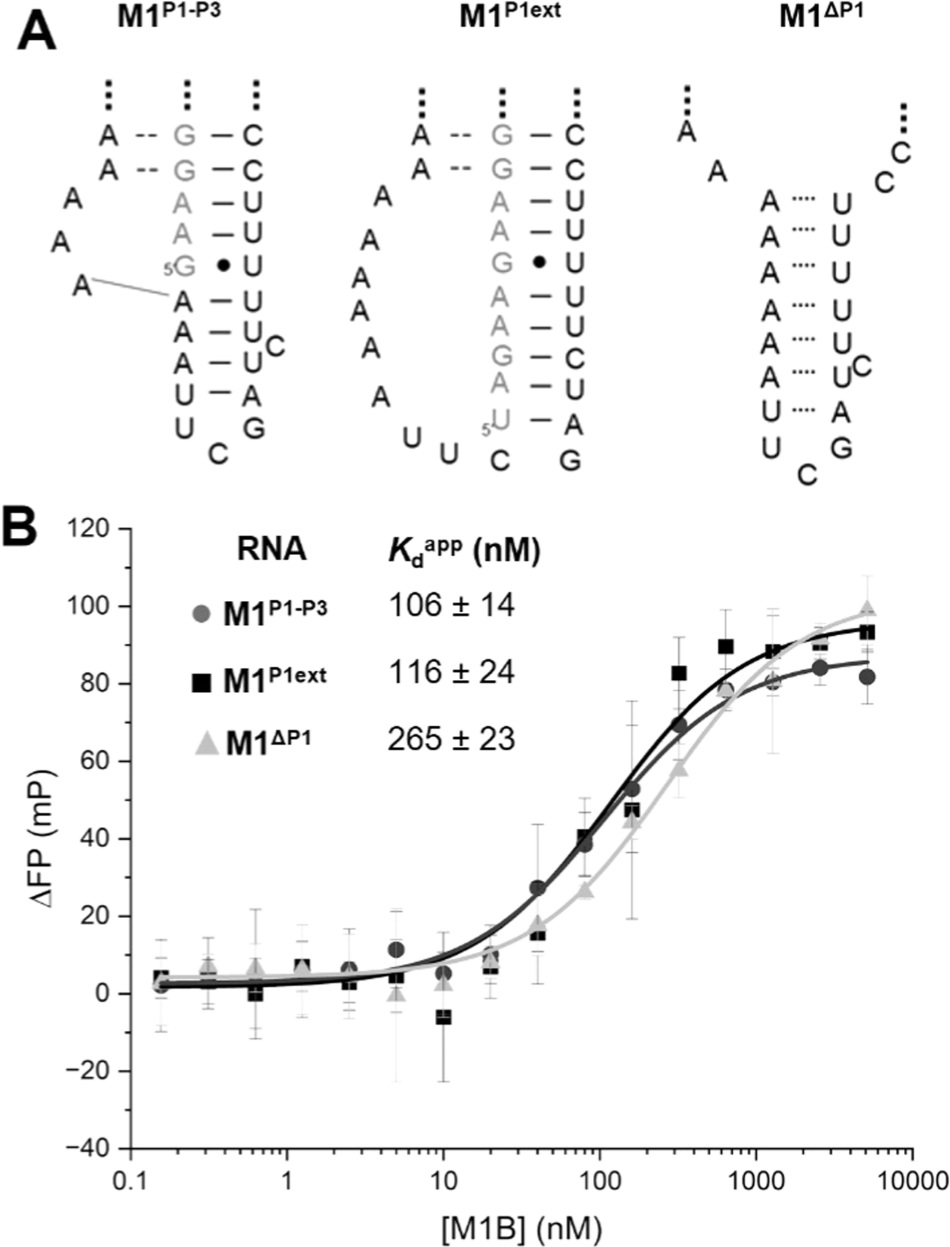
The contribution of peripheral duplex stacking to triple helix formation and stability. (A) Secondary structure depiction of the linker region of bimolecular M1^AB^ RNA constructs with M1^A^ RNAs (light gray) and M1^B^ RNAs (black). Secondary structure diagrams of the three constructs are contained in Figures S1 and S7. The three constructs are denoted as M1^P1–P3^, M1^P1ext^, and M1^ΔP1^. M1^P1–P3^ contains the wild-type linker. M1^P1ext^ has an extended P1 stem with an insertion of 5 nucleotides. M1^ΔP1^ has a deletion of 5 nucleotides, thereby removing the P1 stem, which may result in the formation of putative base pairs (dotted lines). (B) Fluorescence polarization binding assay results for the M1^AB^ constructs: M1^P1–P3^ (dark gray circles), M1^P1ext^ (black squares), and M1^ΔP1^ (light gray triangles). All binding experiments were conducted in 0.1 mM MgCl_2_, 25 mM NaCl, 25 mM KCl, and 20 mM HEPES, pH 7.4. The apparent *K*_d_ (*K*_d_^app^) is denoted for each construct. The data shown is an average of 5 trials for M1^P1–P3^ and M1^P1ext^ and 4 trials for M1^ΔP1^.

Our mutation and ligation results demonstrate the presence of the P3 helix, which stacks beneath the P1 helix. We next turned to fluorescence polarization to directly evaluate the importance of the triplex–P1-P3 coaxial stacking interactions. These coaxial stack interactions directly connect the triplex region to the peripheral elements. We designed three bimolecular triplex-forming constructs of M1^TH^ with variations in the P1–P3 coaxial stack and connectivity to the triplex region to investigate its impact on triple helix stability (Figures 5A, S1, and S7). The constructs were designed based on the M1^AB^ construct previously used to characterize the role of peripheral structural elements on the M1^TH^ stability.^16^ The M1^AB^ constructs adopt the wild-type triplex region interactions and incorporate variations in the P1 and linker regions (Figures S1 and S7). Briefly, the constructs were designed to have variable lengths in the M1^A^ RNA,^16^ the effects of which would impact the P1–P3 stack. Each of the constructs utilizes the same M1^B^ RNA,^16^ which contains the wild-type linker region.

The first construct, M1^P1–P3^, maintains the WT stacking and base pairing of the P1 and P3 peripheral regions. In the second construct, M1^P1ext^, we introduce an elongated P1 stem by adding 5 nucleotides to the M1^A^ RNA that base pair with a portion of the linker sequence in the M1^B^ RNA, thereby mimicking the P1–P3 helical stack. Additionally, this construct has an inserted G akin to the M1^LinkG^ mutation. However, unlike the M1^P1–P3^ construct, where stability is based on the direct helical stacking of the bisected P1 to P3 stem, in the M1^P1ext^ construct, the stacking interactions are due to a single elongated stem. The third construct, M1^ΔP1^, was designed with a deletion of the first 5 nucleotides in M1^A^ RNA, resulting in the removal of the P1 stem. Consequently, this construct does not form the wild-type P1–P3 stack; rather, it can putatively form a stem of 7 base pairs separated from the triplex region by an internal loop (Figure 5A). To demonstrate that the 3 constructs formed triple helices, we performed fluorescence electrophoretic mobility shift assay (fEMSA) experiments at increasing concentrations of M1^B^ RNA. The formation of a new product that migrates slower on a denaturing gel is indicative of triple helix formation (Figure S7).

To quantitatively assess the binding affinity of the M1^A^ molecules of differing lengths to M1^B^, we designed an FP binding assay. In this assay, we titrated M1^B^ RNA while maintaining constant concentrations of the respective M1^A^ RNAs. As the M1^AB^ triple helix forms, it is expected that the increased size of the complex will result in an overall slower tumbling motion. This motion can be detected by monitoring fluorescence polarization. In this assay, the associated complex representing the bound state of M1^A^ RNAs, has a more polarized emission when compared to the unbound M1^A^ RNAs. To determine the binding affinity of the M1^AB^ triplex formation, we plotted the change in polarization between the bound and unbound states of the M1^A^ RNAs against the M1^B^ concentration (Figure 5B). The dissociation constant for the 3 constructs was calculated from this plot.

The apparent *K*_D_ of the M1^P1–P3^ construct was determined to be 106 nM, while that of the M1^P1ext^ construct was 116 nM. The difference in the binding affinity of these two constructs was not significant; their apparent *K*_Ds_ were within experimental error. A comparison of the linker regions of the two constructs shows a potential structure characterized by extended stacking interactions in the P1 linker region of both constructs. In the M1^P1–P3^ construct, we have 9 stacks, while in the M1^P1ext^ construct, we have 10 continuous stacks. In both cases for M1^P1–P3^ and M1^P1ext^, the extended stacking of the duplex region is directly connected to the triplex region. This accounts for the tight binding, which indicates the formation of a stable triple helix. In contrast, removal of the P1 stem results in an apparent *K*_*D*_ of 265 nM. When compared to the M1^P1–P3^ construct, the *K*_*D*_ of the M1^ΔP1^ increases 2.5-fold. This M1^ΔP1^ construct putatively forms 7 base pairs in the linker region, but the putative helix lacks direct connectivity to the triplex region due to the internal loops (Figure 5); this loss of direct connectivity is likely responsible for the reduced binding affinity. These FP results support both the likelihood of the presence of structure within the linker region of M1^TH^ as well as the importance of direct connectivity of peripheral elements to the triplex region.

### Full-length Structural Model of M1^TH^

Our results described above strongly suggest that the M1^TH^ linker forms an ordered structure, specifically that it adopts a helical conformation. In an effort to model the full-length M1^TH^ structure with the newly identified P3 helix, we first sought to model the P3 helix using a combination of threading, Monte Carlo simulation, and manual fitting (**Methods**). Due to the complex geometry involved in creating a helical conformation while preserving the maximum number of crystallographic contacts (Figure S4), traditional single nucleotide ab initio modeling of the linker using, for example, the Rosetta software suite^25^ was not possible.

**Figure 6:**
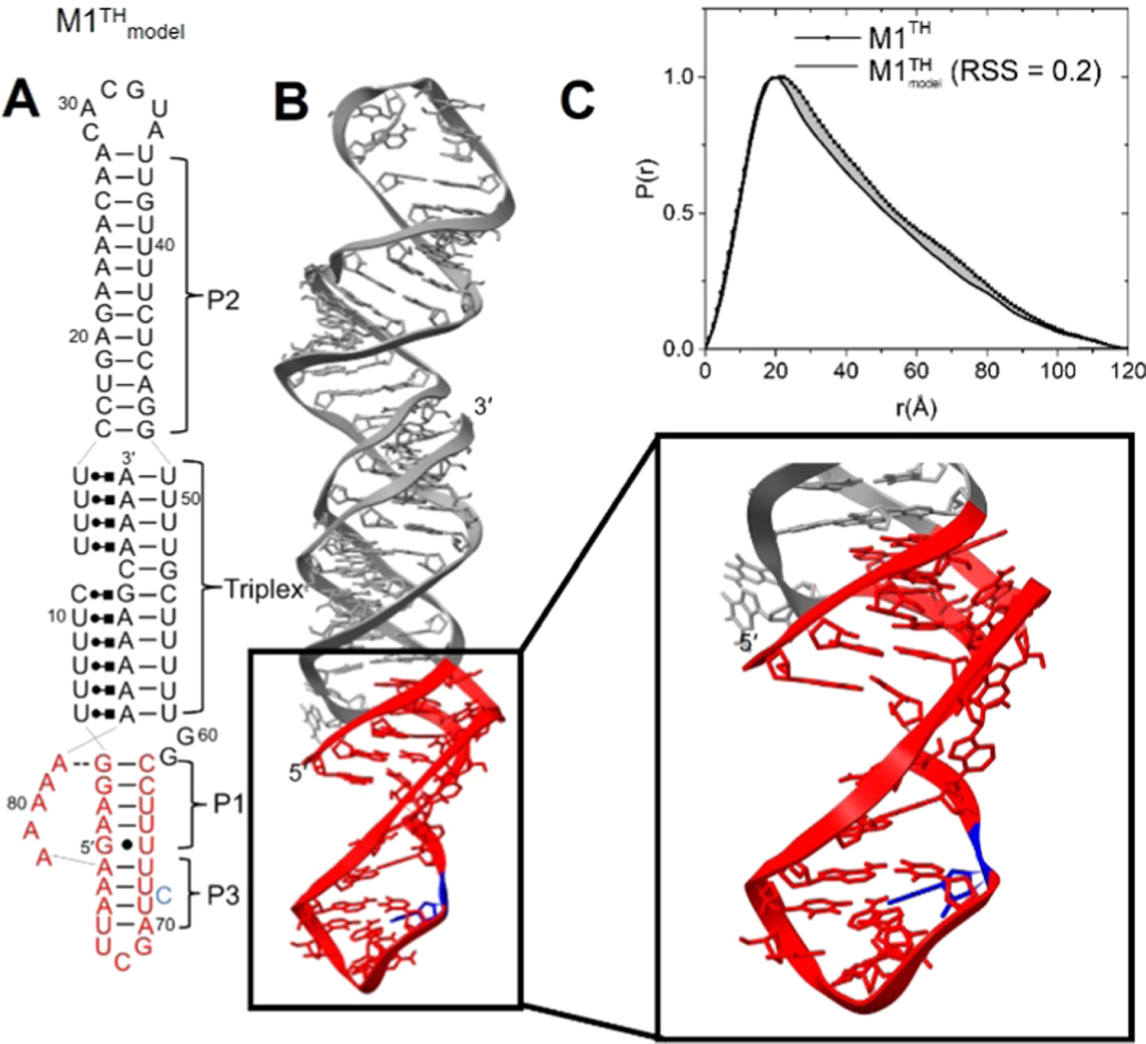
Model of the full length M1^TH^ structure. (A) Secondary structure of the full-length M1^TH^ (M1_^TH^model_) with the P3 helix indicating base pairs A77-U66, A76-U67, A75-U69, U74-A70 in red and bulged residue C68 in blue. (B) A molecular model of the full-length MALAT1 triple helix (M1^TH^_model_). The P1–P3 coaxial stack is colored red and the bulged residue in the P3 helix is colored blue. (C) Comparison of the experimental M1^TH^ SAXS *P*(*r*) plot (circles + line) with the *P*(*r*) plot calculated from the M1^TH^_model_ (line). The gray shaded area represents the minor differences between our model and the experimental data (RSS = 0.2).

Backbone threading is a useful structural tool during the modeling process when there is a known conformation but no existing target structure. The cyclic dimeric guanosine monophosphate riboswitch (cdGR), solved by X-ray crystallography (PDBID: 3IWN),^27^ contains a region consistent with the base pair configuration shown in Figure 6A, namely two base pairs separated by a single nucleotide bulge. This “helical bulge” conformation serves as the threading template for nucleotides 66–70 and 74–77 of M1^TH^ (**Methods**) (Figure S8). While this exercise provides molecular coordinates for our structural model, it also induces a geometrical limitation in connecting A_77_ of the basal helix to A_83_ of the 3′ tail, where five nucleotides need to span >25 Å. An energetically favorable stack of five adenosine nucleotides covers approximately 21.2 ± 0.3 Å of coordinate space measured as an end-to-end distance (03′ to P). The distance was increased to ∼28 Å by rotating the backbone dihedral of A_81_ by 110° with simultaneous geometry optimization of all atoms within a 6 Å radius, and the resulting structural model was energy minimized (**Methods**).

The final structure of our M1_^TH^model_, shown in ribbon representation in Figure 6B, preserves one A-minor contact between G_5_-A_82_. Nucleotides A_78_, A_79_, and A_80_ are unpaired and traverse a 10 Å gap between A_77_ and A_81_. The remaining 12 nucleotides are shown in Figure 6B to be involved in a P3 helix, demonstrating a highly structured linker region is geometrically possible (see Supporting Information). The global geometry depicts a 33 base pair helical stack defined by the P3–P1–triplex–P2 regions. The *P*(*r*) calculated from a theoretical scattering curve of this wild-type M1^TH^_model_ (**Methods**), is in very close agreement (RSS = 0.2) with experimental SAXS measurements of wild-type M1^TH^ (Figure 6C).

## Discussion

The MALAT1 triple helix-containing RNA is a 94-ntstructural element residing at the mature 3′ end of the lncRNA. It is responsible for protecting the entire transcript from 3′-5′ exonucleolytic cellular degradation machinery. Several previous studies evaluating the function of M1^TH^ have focused on evaluating mutations within the triplex region and the immediately proximal duplex base pairs.^5−7,11^ Mutations within the triplex region or the immediately adjacent base pairs significantly alter the stability of the triple helix.^5,7^ However, the role of the peripheral elements has been underappreciated. Our previous research suggested the importance of peripheral elements in stabilizing the triple helix.^16^ A deletion of the linker region or the P2 stem resulted in an approximately 12 °C difference in thermal melting temperature, thus indicating the importance of these structural elements in the overall triple helix stability. However, the constructs utilized in that study were bimolecular, leaving open the question of whether the quantitative reduction in triple helix stability was due primarily to a reduction in duplex stacking interactions or to bisection of the unimolecular RNA into a bimolecular construct. For this reason, in this study, we evaluated the role of the linker sequence utilizing unimolecular structures of M1^TH^.

### Uncovering the Hidden P3 Linker Helix

Herein, we assess the role of the peripheral linker in the protective mechanism of the triple helix using mutational analyses coupled with structural and functional analyses. The correlation between thermal stability and protection from exoribonucleolytic degradation demonstrated that triplex-mediated functional stability is transmitted, at least in part, from the periphery to the core of the triple helix. In particular, our results show that this stabilization effect may be dependent on the sequence composition. Mutational analysis using the M1^LinkG^ mutant indicates that the dependence on sequence composition is a result of secondary structure formation within the linker region (Figure 3, M1^LinkG^). The formation of this linker helix structure is further demonstrated by the ligation of the M1^truncA77^ construct (Figure 4) and the binding affinities measured using fluorescence polarization (Figure 5). Global measurements of the *R*_g_ and *P*(*r*) from SAXS experiments together with molecular modeling of the wild-type M1^TH^ support an extended rod-like structure, wherein an ordered P3 helix forms a helical stack with the P1-triplex-P2 tertiary structure (Figures 2 and 6). Given that the linker is at least 43 Å from the 3′ end, our results indicate that intramolecular interactions within the linker act at a distance to influence the 3′ end stability.

Uncovering the P3 linker helix was surprising given that the linker sequence is not highly conserved across MALAT1 sequences.^7^ Additionally, constructs having a truncated linker were demonstrated to be functional using an intron-less β-globin mRNA reporter.^5,6^ While those truncated linker constructs, lacking the P3 helix, have been shown to lead to functional accumulation in cells, recent studies have demonstrated that the protective function of the triple helix in cells may be enhanced by a protein binding partner.^1,9,11,17^ Our results indicate that the inherent stability and function of the triple helix *in vitro* is enhanced by the P3 helix (Figure 3B,C).

The discovery of novel functional RNAs inevitably requires defining a minimal functional sequence. However, for several RNAs, studies based on truncated constructs have produced results inconsistent with full-length constructs.^27,33−38^ The well described case of the minimal and full-length hammerhead ribozyme structures revealed the deleterious structural effects of truncating the ribozyme.^39^ Early minimal structures did not agree with biochemical studies.^33,38^ Later studies of the ribozyme demonstrated the presence of contacts between distant stems that primed the enzyme for catalysis, contributing to a 1000-fold enhancement in enzymatic activity.^34^ Similar detrimental functional results arose from defining a truncated minimal structure of the cyclic di-GMP riboswitch. In contrast with earlier reports by Sudarsan et al., in which a minimal structure of the riboswitch bound the ligand with a *K*_*d*_ of 1 nM,^35^ a full-length structure of the riboswitch was shown to bind the ligand as much as 100-fold tighter.^36^ In our present study, the identification of the new structural element, the P3 helix, in the MALAT1 triple helix-containing RNA increases its thermostability and functional stability and is thereby consistent with the studies of the minimal and full-length constructs of the hammerhead ribozyme and the cyclic di-GMP riboswitch.

**Figure 7:**
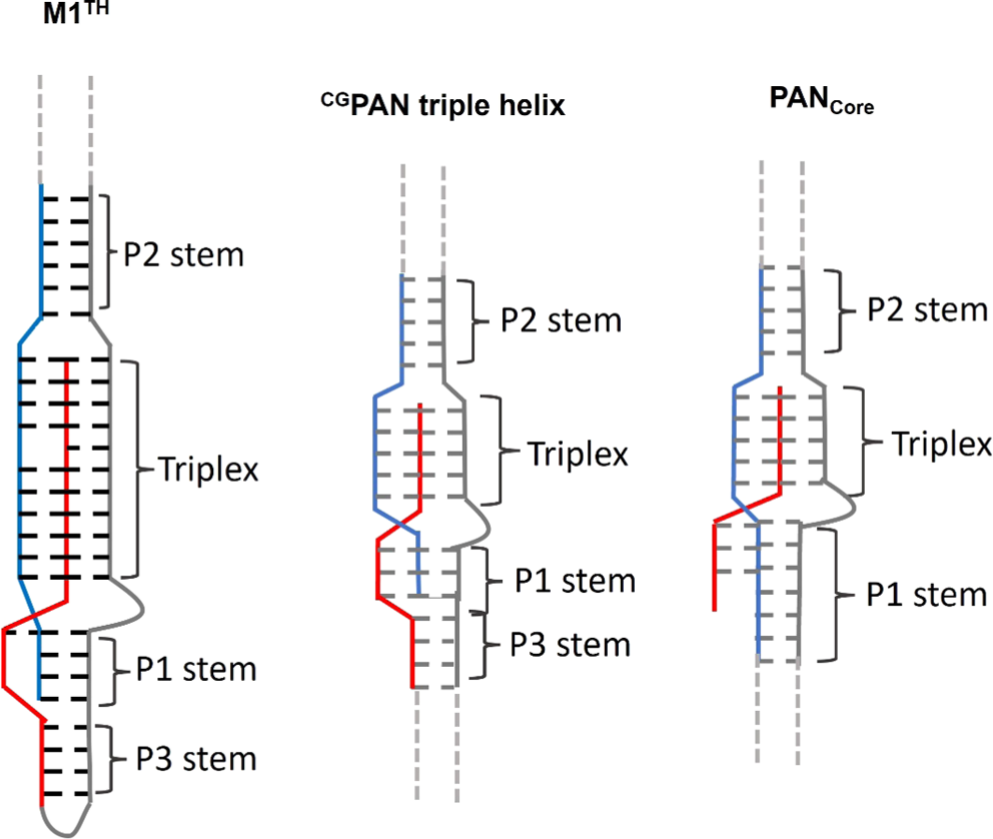
Depiction of the secondary structures of the MALAT1 and the PAN triple helices, showing the anchoring and direct connectivity of the peripheral element paired stem regions. The pyrimidine-rich Hoogsteen strand is in blue, the purine-rich strand is in red, and the pyrimidine-rich strand is in gray. Dotted lines indicate the residues not shown. The M1^TH^ and ^CG^PAN triple helices have similar anchoring of the Hoogsteen and purine-rich strands to peripheral elements P1 and P3, respectively. The binding affinity of the ^CG^PAN triple helix was previously reported to be 74-fold tighter than that of the unanchored PAN_Core._^19^

The connectivity of the triplex to the surrounding duplexes is important for overall RNA stability. In the structure of the MALAT1 triplex, the Hoogsteen triplex strand is directly connected to the adjacent P1 helix (Figure 7, blue strand), and the purine-rich, poly-A-rich tail strand is directly connected to the P3 helix (Figure 7, red strand). These triplex–duplex connections anchor the triple helix structure, leading to an increase in stability. Our fluorescence polarization binding results demonstrate that direct connectivity of the peripheral duplex to the triplex increases the binding affinity of bimolecular constructs by 2.5-fold (Figure 5, M1^P1–P3^ vs M1^ΔP1^). This is consistent with prior mutation studies conducted by Brown et al., in which they showed that the loss of the starting base pairs that connect the duplex to the triplex resulted in decreased accumulation of the RNA in cells.^5^

This notion of the functional importance of direct anchoring connectivity between the triplex region and peripheral duplex is further demonstrated by a recent study of the PAN triple helix-containing RNA.^19^ In that study, a bimolecular PAN triple helix construct, ^GC^PAN, in which both the Hoogsteen and the A-rich tail strand were anchored to paired regions resulted in a 74-fold improvement in binding affinity compared to the PAN_Core_ construct, which lacked similar anchoring (Figure 7). These studies demonstrate that the overall stability of the triple helix is directly related to the connectivity of the three strands of the triple helix to peripheral elements.

### Structural and Functional Stability Conferred by Extended Stacking Interactions

Here we have presented a model of the full-length MALAT1 triple helix-containing RNA comprising 33 layers of intramolecular stacking interactions, which confer structural stability to the 3′ terminus and support evasion of exonucleolytic degradation pathways. In this full-length structural model of M1^TH^, which adopts an extended rod-like structure, the long coaxial stack involves 13 base pairs of the apical P2 duplex coaxially stacked onto 11 stacking interactions within the triplex region, which is coaxially stacked onto 9 base pairs of the P1–P3 helices (Figure 6). This continuous stacking in this RNA is intermediated by a 2-nucleotide bulge between the triplex region and P1 stem.

Extended intramolecular and intermolecular coaxial stacking interactions are a common mechanism by which structural and functional stability are conferred on RNA molecules. Long-range functional communication is facilitated by stacking interactions. For example, protein synthesis on the ribosome is supported by structural stabilization achieved through intramolecular coaxial stacking interactions of up to 70 layers.^40^ Additionally, the structural stabilization required for direct readout of tRNA aminoacylation status by the T-box riboswitch is achieved through an extended intermolecular, 29-layered coaxial stack between the T-box and tRNA.^41^ Similarly, we demonstrate a strong intramolecular, 33-layered coaxial stack in M1^TH^ RNA, which facilitates the anchoring of the triplex strand and functional stability.

We describe a new duplex structure, P3, formed in the linker region of M1^TH^. Our results demonstrate that triple helix stability is enhanced due to the direct anchoring of the triplex region to peripheral elements. Given this enhancement, we propose that peripheral elements may be a mechanism by which other naturally occurring or engineered RNA triple helices can also be stabilized. Such interconnectivity of triplex regions and peripheral elements may also be important for the function and stability of intermolecular RNA-DNA triple helices involved in biological regulatory processes.

## Supporting Information Available

Figure S1. Secondary structure depictions of various mutants, Table S1. and Table S3. Melting temperatures of various mutants, Table S2. Experimental and theoretical SAXS data, Figure S2. and Figure S5. Melting profiles of the various mutants, Figure S3. Time-course degradation gels for different linker mutants, Figure S4. Ribbon representation of mutants, Figure S6. Ligation assay of radioactive mutant, Figure S7. Triple helix structures formed by bimolecular constructs of M1^TH^, Figure S8. C-di-GMP riboswitch structure, a description of fEMSA experimental details, and a description of the limitations of the full length M1^TH^ model (PDF)

## Abbreviations

LncRNA: long noncoding RNA
MALAT1: metastasis-associated lung adenocarcinoma transcript 1
M1^TH^: MALAT1 triple helix-containing RNA
SAXS: small-angle X-ray scattering
RNAP: T7 RNA polymerase
SEC: size-exclusion chromatography
DSF: differential scanning fluorimetry
FFT: fast fourier transform
FP: fluorescence polarization
PAGE: polyacrylamide gel electrophoresis
DEPC: diethyl pyrocarbonate
cdGR: cyclic dimeric guanosine monophosphate riboswitch
MENβ: multiple endocrine neoplasia-β
Rnl2: RNA Ligase 2
fEMSA: fluorescence electrophoretic mobility shift assay
*K*_D_: dissociation constant
