# Supplementary material for: A Newly Identified Peripheral Duplex Anchors and Stabilizes the MALAT1 Triplex
Source: Biochemistry. 2024 Aug 27;63(18):2280–92. doi: 10.1021/acs.biochem.4c00156 (PMC11411715; doi:10.1021/acs.biochem.4c00156)
Supplement: Supplementary file 1 — bi4c00156_si_001.pdf [file bi4c00156_si_001.pdf]

Supplementary information

A newly identified peripheral duplex anchors and stabilizes the MALAT1 triplex.

AUTHOR NAMES

Mary N. Mwangi[†1], *Michael J. Yonkunas*[†1,2], *Abeer A. Ageeli*[†1,3], *Kayleigh R. McGovern-Gooch*[1,4], *Sevde Yilmaz*[1], *and Nathan J. Baird*[1]*

[†] *M.N.M, M.J.Y, and A.A.A contributed equally to this work.*

** Correspondence should be addressed to N.J.B. at nbaird@sju.edu*

AUTHOR ADDRESS

[1] M.N.M and N.J.B: Department of Chemistry & Biochemistry, Saint Joseph's University, 600 S. 43rd Street Philadelphia, PA, 19104, USA.

[2] M.J.Y: Discovery Chemistry Research and Technologies, Lilly Research Laboratories, Eli Lilly and Company, Indianapolis, IN, USA

[3] A.A.A: Department of Physical Sciences, Chemistry Division, College of Science, Jazan University, P.O. Box. 114, Jazan 45142, Kingdom of Saudi Arabia

[4] K.R.M: Arbutus Biopharma, Warminster, PA, USA

Supplementary Figures and Tables.

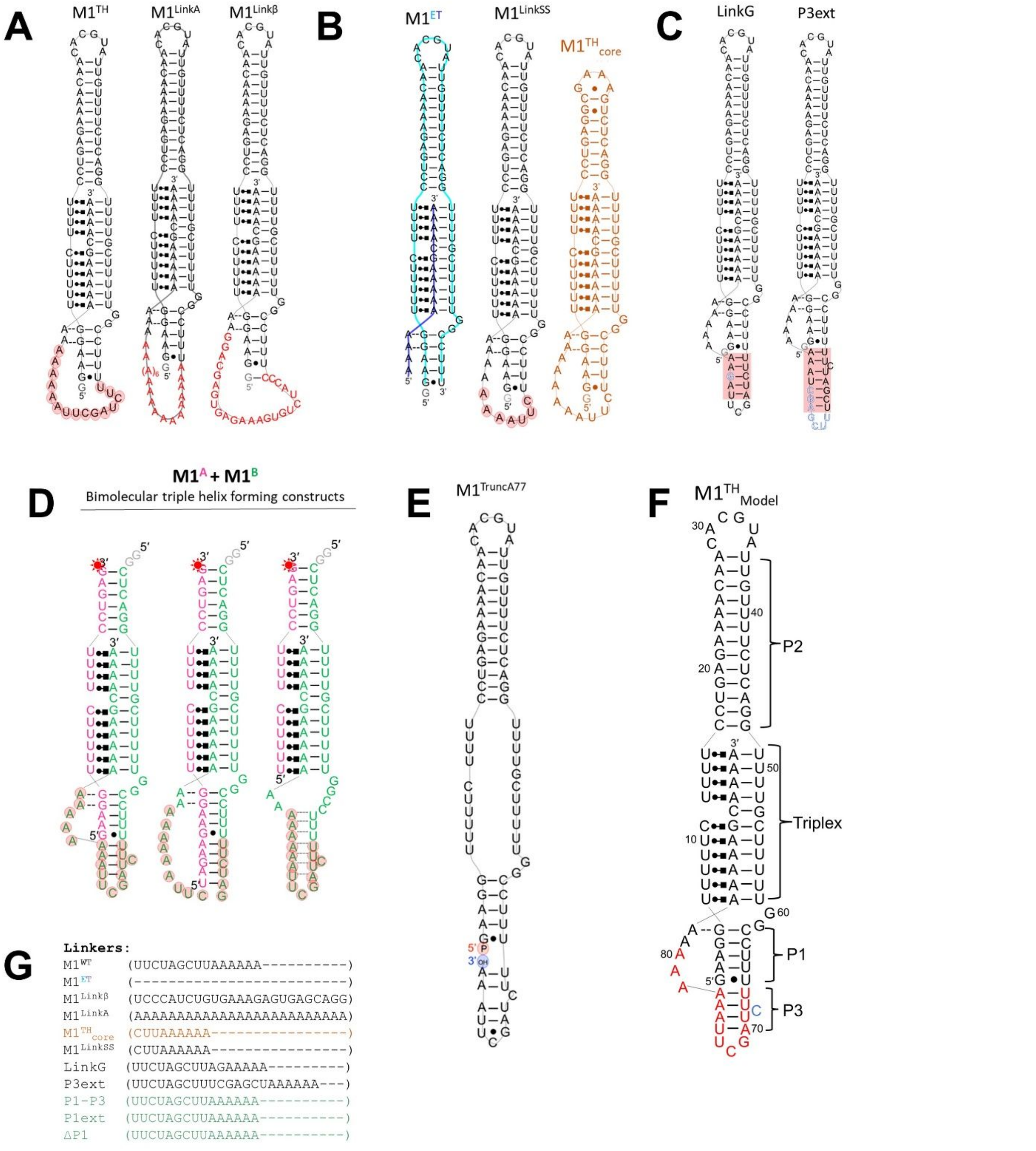

Figure S1. Side-by-side comparison of the secondary structures of the mutants used in this study.

A) Structures of the wild type MALAT1 triple helix (M1TH), M1^{LinkA} mutant and M1$^{Link\beta}$ mutant

described in **Figure 1. B)** Structures of bimolecular construct $M1^{ET}$, unimolecular constructs $M1^{LinkSS}$ and $M1^{TH}_{Core}$. $M1^{ET}$ and $M1^{LinkSS}$ constructs are described in **Figure 2**. $M1^{LinkSS}$ was derived from $M1^{TH}_{Core}$ [1]. **C)** Structures of unimolecular constructs LinkG and P3ext described in **Figure 3**. **D)** Structures of the 3 bimolecular constructs formed by the association of the $M1^{A}$ RNA (pink) with the $M1^{B}$ RNA (green). The 3 constructs are designated P1-P3, P1ext and ΔP1, they are described in **Figure 5**. **E)** Secondary structure depiction of the $M1^{truncA77}$ construct used in the ligation assays and described in **Figure 4**. **F)** Secondary structure of the $M1^{TH}_{Model}$ described in **Figure 6**. **G)** A comparison of the linker sequences of all the described constructs.

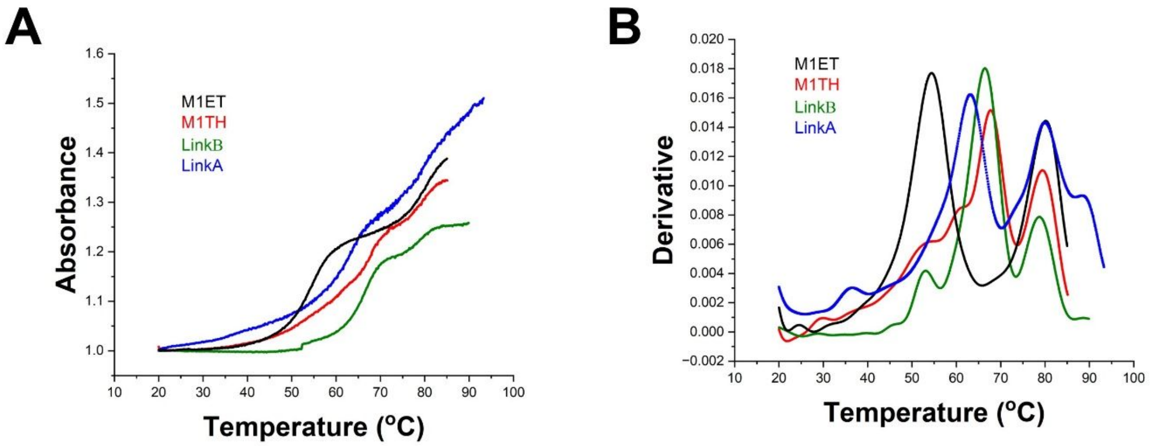

Figure S2: The effect of the linker sequence and length on $M1^{TH}$ stability using UV melt assay of different linker mutants. A) Overlay of the raw absorbance signal of the different linker mutants shown in black, red, blue, and green for $M1^{ET}$, $M1^{TH}$, $M1^{linkA}$, and $M1^{link\beta}$, respectively. **B)** Overlay of the first derivative of the raw absorbance signal of the linker mutants in **A** showing the two peaks for melting tertiary and secondary structures. Some noise is present in the data as observed in the small peaks (e.g. around 35 °C). Despite this noise, the key finding of two dominant peaks for all RNA constructs, corresponding to triplex and secondary structure melting, remains clearly evident.

Table S1. A comparison of the tertiary and secondary melting temperatures of $M1^{TH}$, $M1^{ET}$, $M1^{Link\beta}$, and $M1^{LinkA}$ obtained by UV melt assays in 1 mM $MgCl_2$, 25 mM NaCl, and 25 mM KCl in 20 mM HEPES, pH 7.4, obtained in triplicate. $T_{m,1}$ depicts triplex melting while $T_{m,2}$ depicts the secondary structure melting temperature. The error shown is the standard deviation of the 3 trials.

RNA	$T_{m,1}$ (°C)	$T_{m,2}$ (°C)
$M1^{ET}$	54.1 ± 0.1	80.3 ± 0.1
$M1^{TH}$	66.7 ± 0.1	78.2 ± 0.2
$M1^{Link\beta}$	65.7 ± 0.3	79.3 ± 0.6
$M1^{LinkA}$	63.1 ± 0.3	79.4 ± 0.8

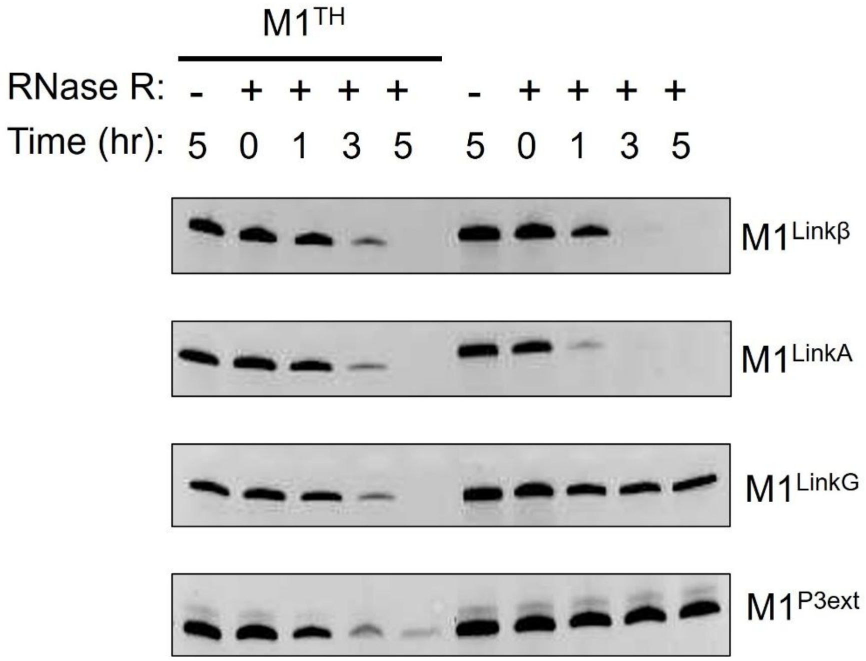

Figure S3. Time-course degradation of each linker mutant in comparison to M1TH that is run on the same gel (Figure 1D and Figure 3C). All the linker mutants are degraded faster than M1TH, with the exception of the stabilizing linker mutants M1^{LinkG} and M1^{P3ext}, which show maximal protection from RNase R activity even after 5 hr. Differential degradation of M1TH is seen when different batches of RNase R are used. This is seen in the degradation of M1TH in the last gel panel when compared to the first 3 gels. However, even with this differential degradation, we observe significant degradation of M1TH within 5 hours. For M1^{P3ext} gel the doublet seen in the samples is due to an aberration during gel running.

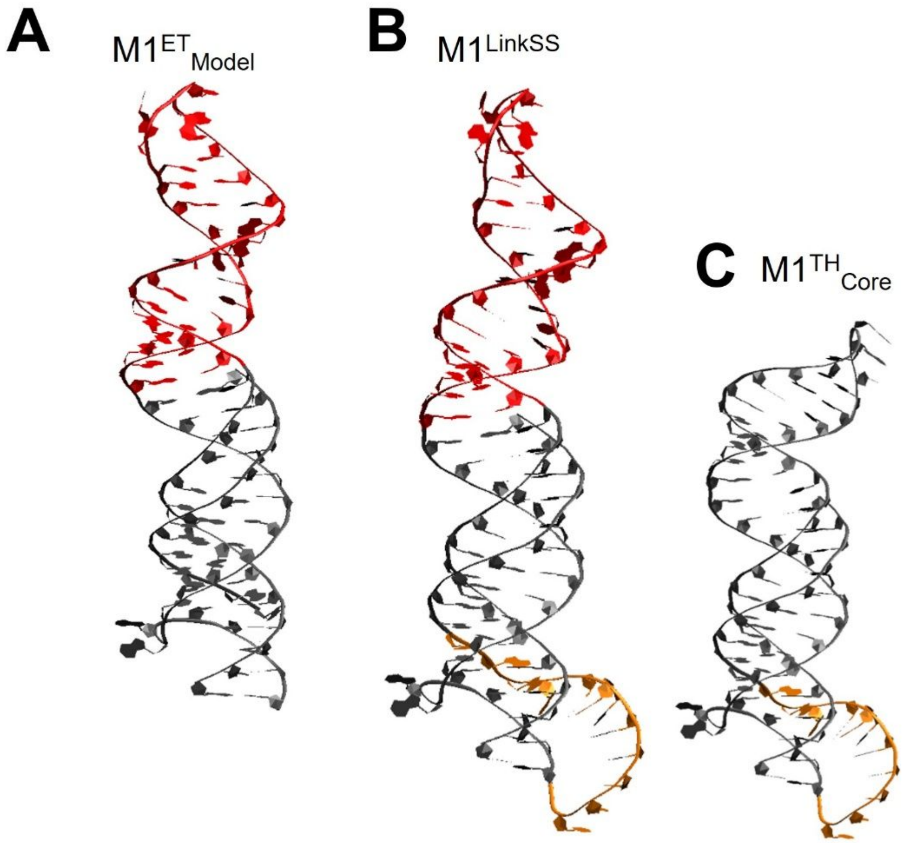

Figure S4. A) A ribbon representation of the $M1^{ET}_{Model}$, a two-strand model consisting of the crystal core construct (shown in grey) with the wild-type P2 helix (shown in red) and the linker region removed (see secondary structure in **Figure 2B and Figure S1**). **B)** A ribbon representation of the $M1^{LinkSS}$, a single-strand model consisting of the crystal core construct (shown in grey) with the wild type P2 helix (shown in red) and the truncated linker region of the crystal core (shown in orange). **C)** A ribbon representation of the $M1^{TH}_{Core}$ crystal structure (PDBID:4PLX) where the truncated linker is highlighted in orange.

Table S2. Experimental and calculated SAXS parameters R_g and D_{max} (see **Methods**). Error reported in parenthesis.

Experimental			Calculated		
**RNA**	***R*$_g$ (Å)**	***D*$_{max}$ (Å)**	**Model**	***R*$_g$ (Å)**	***D*$_{max}$ (Å)**
**$M1^{TH}_{Core}$**	N/A	N/A	**$M1^{TH}_{Core}$**	24 (1)	83 (6)
**$M1^{TH}$**	30.7 (0.6)	128 (11)	**$M1^{TH}_{Model}$**	31 (1)	119 (4)
**$M1^{ET}$**	26.6 (0.5)	102 (8)	**$M1^{ET}_{Model}$**	27 (1)	99 (3)
**$M1^{LinkSS}$**	N/A	N/A	**$M1^{LinkSS}_{Model}$**	28.5 (1)	105 (5)

Table S3. A comparison of the secondary and tertiary melting temperatures of $M1^{TH}$, $M1^{LinkG}$, and $M1^{P3ext}$ obtained by differential scanning fluorimetry assay in 25 mM NaCl and 25 mM KCl in 20 mM HEPES, pH 7.4, and varying $MgCl_2$ concentration. Experiments were done in triplicate, and the error shown depicts the standard deviation. $T_{m,1}$ corresponds to the triplex melting (bolded) while $T_{m,2}$ corresponds to the secondary structure melting.

[$MgCl_2$] mM	$M1^{TH}$	$M1^{LinkG}$	$M1^{P3ext}$	
0.1	**54.2 ± 1.2**	**57.0 ± 0.1**	**61.3 ± 0.1**	**$T_{m,1}$ (°C)**
	68.7 ± 0.8	68.5 ± 0.1	71.1 ± 0.1	$T_{m,2}$ (°C)
0.3	**58.1 ± 0.8**	**60.8 ± 0.4**	**63.0 ± 0.1**	**$T_{m,1}$ (°C)**
	71.0 ± 0.5	72.2 ± 0.1	72.7 ± 0.2	$T_{m,2}$ (°C)
0.6	**61.6 ± 1.5**	**63.8 ± 0.1**	**65.6 ± 0.1**	**$T_{m,1}$ (°C)**
	73.9 ± 0.6	74.9 ± 0.1	74.4 ± 0.2	$T_{m,2}$ (°C)
1.0	**65.6 ± 0.1**	**66.8 ± 0.2**	**67.6 ± 0.2**	**$T_{m,1}$ (°C)**
	76.5 ± 0.6	76.9 ± 0.1	77.8 ± 0.2	$T_{m,2}$ (°C)

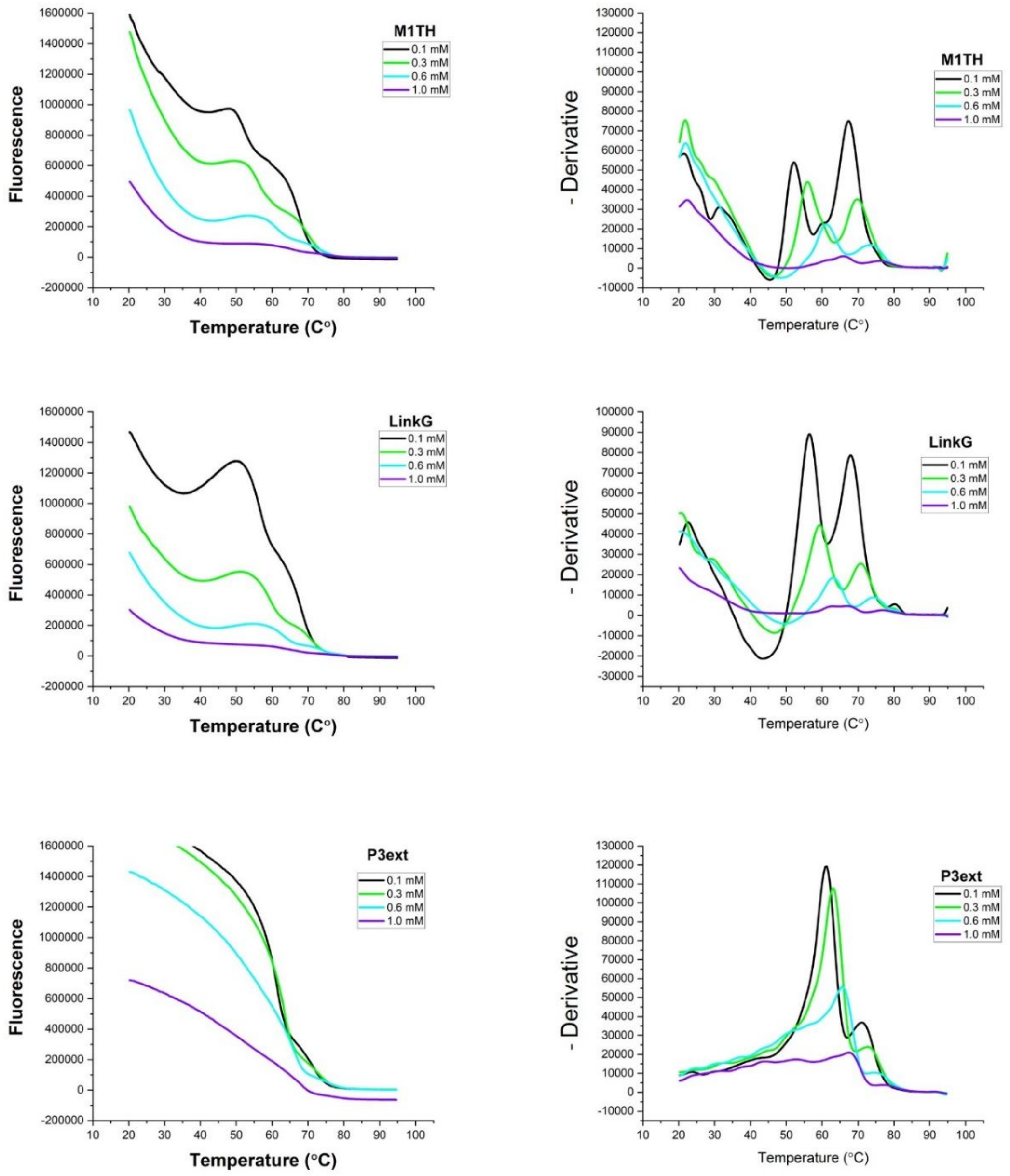

Figure S5: Comparison of the raw signal and derivative melting profiles of M1TH, LinkG and P3ext construct at varying magnesium concentrations obtained by DSF analysis. It is important to note that the data presentation shown overemphasizes the high fluorescence signal seen in the lower magnesium concentrations. At high magnesium concentrations the fluorescence of the dye is

quenched. However, zooming in on the derivative plot for 1 mM magnesium experiments reveals clear peaks readily identifiable by the OriginPro peak finding algorithm.

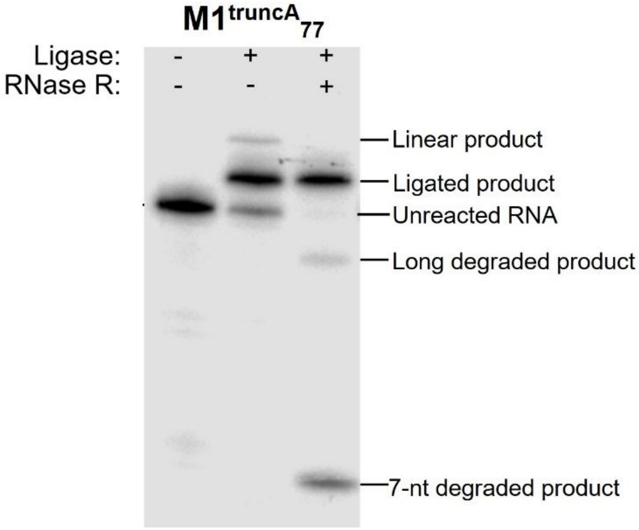

Figure S6. Ligation assay of radiolabeled M1$^{truncA}{}_{77}$ on 12% dPAGE. The addition of RNA ligase 2 (Rnl2) results in the appearance of a new ligated product that migrates slower on the 12% dPAGE than the unreacted one. The formation of ligated circular RNA is confirmed by the addition of RNase R, which results in the degradation of only the linear ligated product and the unreacted RNA. RNase R treatment also produces degraded products of different lengths (long degraded product and 7-nt degraded product).

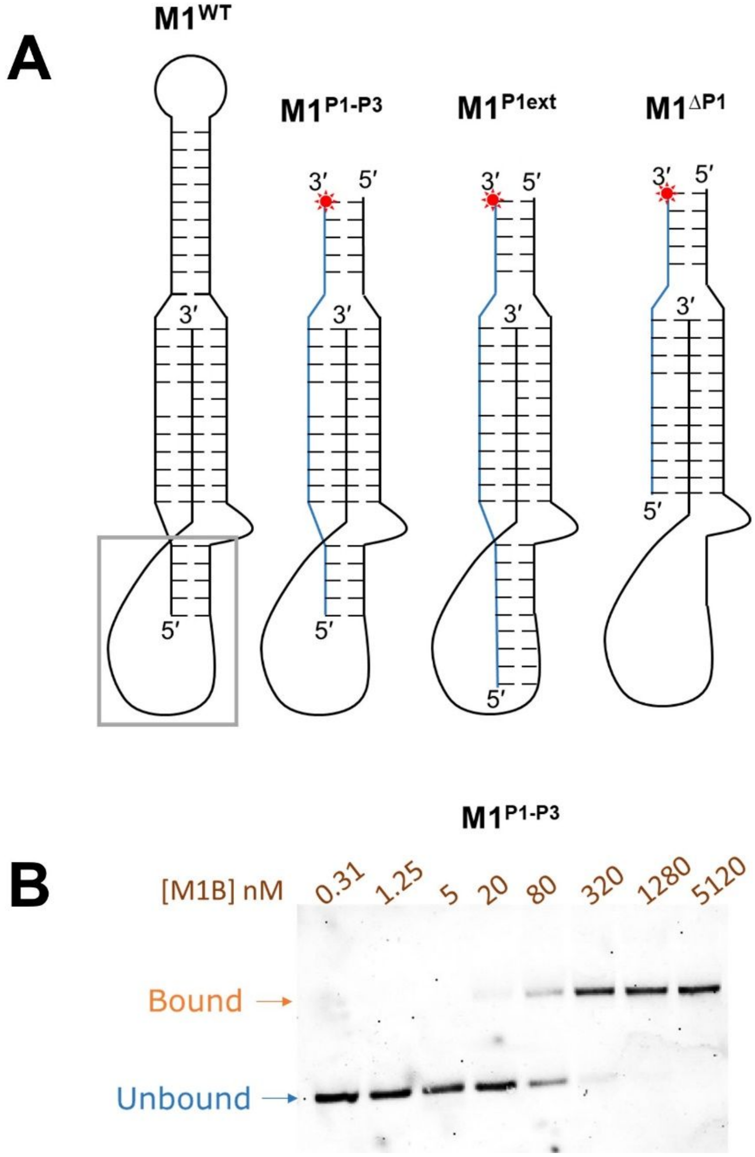

Figure S7. The formation of triple helix structures by different M1TH bimolecular constructs. A. Cartoon depiction comparing the three different bimolecular constructs M1$^{P1\text{-}P3}$, M1^{P1ext}, and M1$^{\Delta P1}$ to the wild type M1TH. The 3 constructs are formed by the association of the M1^{A} RNA (blue) with the M1^{B} RNA (black). The M1^{A} RNA is labelled with a fluorophore at the 3′ end. **B.** A representative gel shift assay on a 6% native PAGE depicting the formation of the triplex for the

P1-P3 construct at increasing concentrations of the M1^{B} RNA. Similar gels were run for each of constructs depicted in panel A.

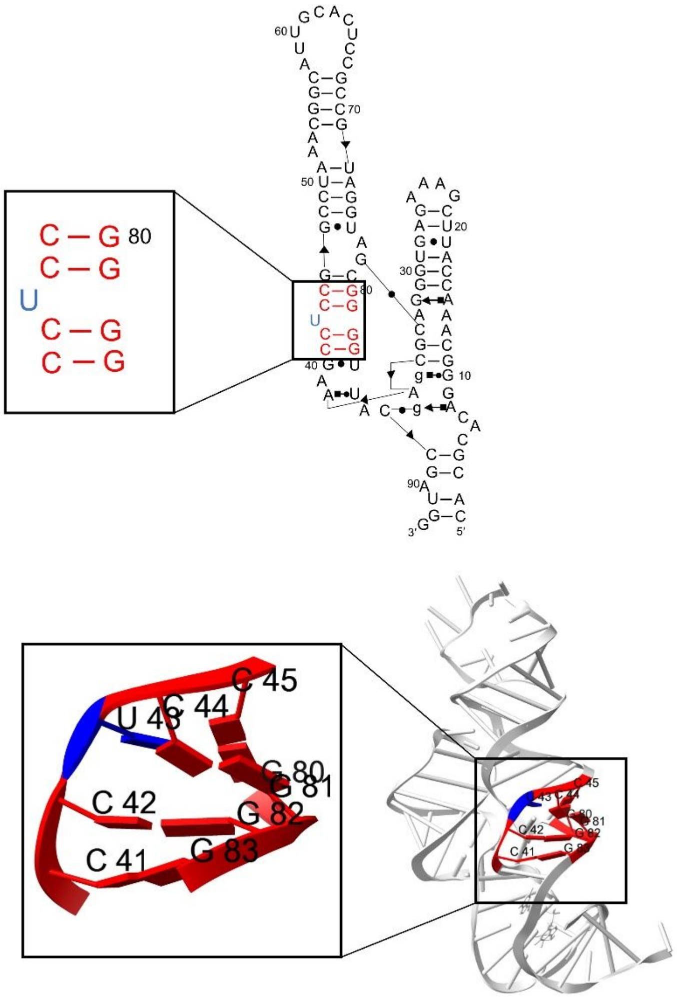

Figure S8. Secondary and tertiary structure of the c-di-GMP riboswitch (PDBID:3IWN). Residues 41-45 and 80-83 (highlighted in red and blue) were used in the threading of P3 when generating the full-length M1TH shown in **Figure 6**.

Supplementary Methods

Fluorescence electromobility shift assay

To determine the formation of triplexes by bimolecular RNA constructs, native polyacrylamide gel electrophoresis (PAGE) mobility shift assays were performed. Eight individual reaction tubes contained one of the 3′-end cy3-labeled $M1^A$ RNAs at a fixed concentration of 8 nM and the unlabelled $M1^B$ at increasing concentrations from 0.31, 1.25, 5, 20, 80, 320, 1280, to 5120 nM. (See **Figure S1** for structure and sequence information). All reactions were prepared in 20 mM HEPES (pH 7.4), 25 mM NaCl, 25 mM KCl, 0.1 mM $MgCl_2$. The samples were incubated at room temperature for approximately 3 hours to facilitate the formation RNA complex, mixed with native gel loading dye (0.1% bromphenol blue, 10% glycerol) and then applied to a 6% native polyacrylamide gel. The gel was run at 25 watts for approximately 30 minutes in 0.5X THE buffer having 0.1 mM $MgCl_2$. Bands containing labelled $M1^A$ RNAs were visualized in an *Amersharm™ Typhoon imager* (Cytiva) using a cy3 filter immediately after electrophoresis. Gel images were analyzed and bands that contained $M1^A$ RNA (unbound and bound) were identified and quantified using *ImageJ*.

Supplementary Information

Limitations of the full length $M1^{TH}$ model

The main limitation of this structural model is the crystallographic templates on which it is based. The "core" structure of $M1^{TH}$ (PDBID: 4PLX) is a stable structure under both crystallographic and certain solution conditions[1–3], although even at 3.1 Å resolution, side chain assignments can be ambiguous[4], for example, the two crystallographic A-minor interactions A_{81} and A_{82}[1]. This is also the case for the 3.2 Å resolution bacterial c-di-GMP riboswitch (PDBID:3IWN)[5] used in the threading of P3.

References

(1) Brown, J. A., Bulkley, D., Wang, J., Valenstein, M. L., Yario, T. A., Steitz, T. A., and Steitz, J. A. (2014) Structural insights into the stabilization of MALAT1 noncoding RNA by a bipartite triple helix. *Nature Structural & Molecular Biology 2014 21:7 21*, 633–640.

(2) Wilusz, J. E., JnBaptiste, C. K., Lu, L. Y., Kuhn, C. D., Joshua-Tor, L., and Sharp, P. A. (2012) A triple helix stabilizes the 3′ ends of long noncoding RNAs that lack poly(A) tails. *Genes Dev 26*, 2392–2407.

(3) Ageeli, A. A., McGovern-Gooch, K. R., Kaminska, M. M., and Baird, N. J. (2019) Finely tuned conformational dynamics regulate the protective function of the lncRNA MALAT1 triple helix. *Nucleic Acids Res 47*, 1468–1481.

(4) Arkhipova, V., Guskov, A., and Slotboom, D. J. (2017) Analysis of the quality of crystallographic data and the limitations of structural models. *Journal of General Physiology 149*, 1091–1103.

(5) Kulshina, N., Baird, N. J., and Ferré-D'Amaré, A. R. (2009) Recognition of the bacterial second messenger cyclic diguanylate by its cognate riboswitch. *Nat Struct Mol Biol 16*, 1212–1217.
